# Online interventions for dementia caregiver burden and self-efficacy: A systematic review and meta-analysis

**DOI:** 10.1016/j.ijnsa.2026.100490

**Published:** 2026-01-22

**Authors:** Qiuxia Li, Dou Zhang, Éle Quinn, Prof. Kieran Walsh, Dr. Sinéad Hynes

**Affiliations:** a Discipline of Occupational Therapy, School of Health Sciences, College of Medicine, Nursing and Health Sciences, University of Galway; b School of Nursing and Midwifery, College of Medicine, Nursing and Health Sciences, University of Galway; c Irish Centre for Social Gerontology, Institute for Lifecourse and Society, University of Galway

**Keywords:** Dementia, Alzheimer's disease, Caregiver burden, Self-efficacy, Mental health, Internet-based intervention

## Abstract

**Background and Objectives:**

There is growing interest in online psychosocial interventions to address burden and self-efficacy among informal caregivers of people with dementia. However, their effectiveness remains unclear, especially given ongoing debates and limited evidence of online delivery modes. We aimed to synthesize evidence on these online psychosocial interventions, highlight the current state of research, and identify priorities for future studies.

**Methods:**

A systematic review with meta-analyses was conducted. We included randomized controlled trials comparing online psychosocial interventions to usual care or waitlist control for burden and self-efficacy. Six electronic databases were searched for studies published over the last two decades. Two reviewers evaluated eligibility, extracted data, and assessed risk of bias and quality of evidence. Patient and Public Involvement, as a means to contextualise the results in the lived experience of caregivers, was employed to aid interpretation of the findings. The review protocol was registered in PROSPERO (CRD42023392232)

**Results:**

Nineteen articles involving 2,264 participants were included. Of these, 15 reported caregiver burden, seven reported self-efficacy, and three studies reported both outcomes. Most interventions were multi-component online psychosocial programs delivered via web-based platforms, mobile applications, or videoconferencing tools. They incorporated asynchronous, synchronous, or blended delivery modes and were delivered either individually or in groups. Core components of the psychosocial intervention included information provision and education, caregiving tasks support, and caregiver self-care. Meta-analysis of 13 studies showed no statistically-significant post-intervention effect on reducing burden (Standardized Mean Difference [SMD]: −0.06, 95 % Confidence Interval [CI]: −0.25, 0.12, *p* =0.50; *I*² = 47 %), yet analysis of three studies indicated an effect on improving self-efficacy (SMD: 0.20, 95 % CI: 0.03, 0.37, *p* =0.02; *I*² = 0 %). The evidence grade for both outcomes was low due to risk of bias, inconsistency, and imprecision of the results.

**Conclusion:**

We suggest that online psychosocial interventions have little to no short-term effect on caregiver burden but may enhance self-efficacy, though the evidence grade remains low. Online psychosocial interventions featured diversity in their design, content, and delivery characteristics. From the findings, we have identified key areas for development of future research within this field, with a particular focus on addressing methodological limitations and ensuring rigorous design. Sustained support incorporating interactive and real-time elements, tailored approaches, and the integration of lived experience throughout the research process should be considered to improve both the effectiveness and relevance of these interventions.

## Abstract (author highlights)

• Online psychosocial interventions can promote positive psychological outcomes for informal caregivers of people with dementia.
• Despite the rapid acceleration in the development and implementation of online psychosocial interventions, there is limited evidence on their impact on caregiver burden and self-efficacy among informal caregivers of people with dementia.
• Online psychosocial interventions showed no statistically-significant reduction in caregiver burden but demonstrated a positive effect on caregiver self-efficacy.
• We have highlighted essential areas for advancing future research in this field, emphasizing the need to enhance methodological rigor and outlining key factors that could contribute to improving the effectiveness of online psychosocial interventions.

**What is already known**

• Online psychosocial interventions can promote positive psychological outcomes for informal caregivers of people with dementia.
• Despite the rapid acceleration in the development and implementation of online psychosocial interventions, there is limited evidence on their impact on caregiver burden and self-efficacy among informal caregivers of people with dementia.

Alt-text: Unlabelled box dummy alt text

**What this paper adds**

• Online psychosocial interventions showed no statistically-significant reduction in caregiver burden but demonstrated a positive effect on caregiver self-efficacy.
• We have highlighted essential areas for advancing future research in this field, emphasizing the need to enhance methodological rigor and outlining key factors that could contribute to improving the effectiveness of online psychosocial interventions.

Alt-text: Unlabelled box dummy alt text

## 1 Introduction

Globally, it remains the case that the majority of people with dementia are cared for by informal caregivers (e.g., family members, friends) at home (Leverton & Pui Kin Kor, 2023; Steenfeldt et al., 2021). While caregiving may enhance a sense of personal accomplishment and family cohesion (Wang et al., 2022), it can also be overwhelming.

Burden and low self-efficacy can pose significant challenges for informal caregivers of people with dementia, hindering their overall health and well-being (Duplantier & Williamson, 2023). Caregiver burden refers to the level of multifaceted strain perceived by the caregiver from caring for a family member or loved one over time (Liu et al., 2020). Caregivers can often feel burden in objective and subjective terms (Cao & Yang, 2020), especially when they do not have or are not provided with the necessary information, resources, and time (Liu et al., 2022). The consequences of caregiver burden include negative outcomes, such as reduced capacity for care provision, diminished quality of life, and deterioration in physical and psychological health. These consequences can affect both the caregiver and the care recipient (Liu et al., 2020). Moreover, objective constraints and subjective perceptions can impede the maintenance and development of self-confidence (Jennings et al., 2015). In the dementia caregiving context, self-efficacy has been conceptualized as the belief in one’s own ability to carry out different activities related to caregiving (Steffen et al., 2019). It is suggested that caregiver self-efficacy acts as a mediator between caregiver variables (i.e., preparedness, income, and social networks) and outcomes of depression and burden (Phongtankuel et al., 2023). Heightened caregiver self-efficacy is associated with lower levels of both depression and caregiver burden (Phongtankuel et al., 2023).

These challenges highlight the critical need for tailored supports and have led to a strong focus on psychosocial interventions as a means to alleviate caregiver burden and to enhance self-efficacy. Despite ongoing debate about the effectiveness of these kinds of supports themselves (Cheng & Zhang, 2020), there has nonetheless been a rapid acceleration in the development and implementation of online psychosocial interventions. Drivers of this development and adoption include increased access to technology, greater flexibility in delivery, cost-effectiveness (Rohrbach et al., 2023), and the long-standing challenges of transportation and scheduling that in-person delivery can present for caregivers (Jiménez et al., 2022). The COVID-19 pandemic has further reinforced this trend in recent years by necessitating the shift to digital platforms for health and social care services, and the increased normalisation of digitalised supports (Zhang & Ma, 2023). Online psychosocial interventions have now become increasingly commonplace reflecting growing provider acceptance, increased consumer willingness to use telehealth, and regulatory changes that have expanded access and reimbursement for virtual services (Baudier et al., 2023; Getachew et al., 2023). Given a burgeoning normative culture of digital participation and that existing constraints on in-person psychosocial interventions are only likely to intensify in the future, the pressure to further develop and adopt online psychosocial interventions is likely to increase. However, there are concerning questions regarding the extent to which current online developments are research led. Significant knowledge gaps remain around the extent and quality of the evidence available on the specific impacts of online psychosocial interventions, as rigorously evaluated through randomized controlled trials.

Existing assessments of evidence on online psychosocial interventions highlight several aspects that could be strengthened. First, while some previous researchers conducting systematic reviews have included studies on online delivery modes, they have included these studies amidst a much broader range of interventions, often combining various remote modalities, such as those delivered solely via telephone or asynchronous DVD/video formats (Deeken et al., 2019; Frias et al., 2020; Leng et al., 2020). Such breadth offers a wider view of remote support but introduces variability that complicates synthesis, since online, telephone, and DVD-based modalities differ substantially in delivery, accessibility, and user interaction. Online interventions typically utilize digital platforms that offer interactive, multimedia content, providing greater flexibility, interactivity, and access to multimedia resources, while telephone-based interventions rely on scheduled voice-based conversation. Similarly, DVD/video formats deliver pre-recorded, static content that is typically accessed offline, resulting in a different user experience compared to the interactive and adaptable nature of online platforms. Second, many reviewers have incorporated a mix of research study designs (e.g., cohort studies, randomized controlled trials) (Egan et al., 2018; Hopwood et al., 2018; Parra-Vidales et al., 2017; Pleasant et al., 2020; Wallace et al., 2021; Waller et al., 2017). This approach offers advantages by broadening the evidence base, improving generalisability, and capturing the diversity of psychosocial interventions. However, such heterogeneity can also limit the ability to conduct standardised assessment and meta-analysis. Third, the target populations within previous systematic reviews are often diverse, including both formal and informal caregivers, as well as individuals caring for people with a variety of different health conditions (Egan et al., 2018; Pleasant et al., 2020). While this improves generalisability, it increases heterogeneity, since formal and informal caregivers differ in their experiences, support systems, training, and perceived burden and self-efficacy (Tay et al., 2022). Fourth, and critically, there have not been any systematic reviews conducted that have meaningfully captured studies published since the advent of COVID-19, despite the rapid growth of online psychosocial interventions during this period. These reviewers have offered valuable insights into the development of psychosocial interventions but leave the overall quality of synthesised evidence insufficient to clearly judge the effectiveness of online psychosocial support. Ultimately, this compromises our understanding of the degree to which such interventions affect caregiver burden and self-efficacy and limits our capacity to identify research gaps and guide future intervention development. In this review, we address these limitations by (i) focusing exclusively on randomised controlled trials of online interventions, (ii) targeting informal caregivers of people with dementia, (iii) specifying that comparator groups were usual care or waitlist control, and (iv) extending the search to include studies published since the COVID-19 pandemic.

Alongside these specific gaps, a broader critique of systematic reviews has emerged, where the resulting knowledge has been sometimes argued to be irrelevant to people's lives due to inappropriate outcomes or mechanisms that don't align with the target group's circumstances and backgrounds (Uttley et al., 2023). This critique can also be framed as a reflection of a broader long-standing concern regarding randomized controlled trial design, where there has been a growing assertion that the perspective of the target group should be incorporated within the study design in some means. Directly involving stakeholders in the development and interpretation of systematic reviews has been proposed as a means to address this issue, enhancing the actual and perceived usefulness of synthesized research evidence (Merner et al., 2021). There has been a growing call to include those with lived experience of targeted challenges within systematic reviews, through Public and Patient Involvement or similar approaches (Pollock et al., 2018; Zhou et al., 2024). Public and Patient Involvement has been found to be integral in shaping the research questions and methods of reviews, ensuring relevance to both patients and practitioners (Barry et al., 2017). Thus far, this involvement strategy has not been implemented in reviews that focus on online psychosocial intervention for dementia caregivers.

To respond to these various gaps and critiques, the aim of this article was to provide a rigorous synthesis of online psychosocial intervention for informal caregivers of people with dementia, with particular attention to caregiver burden and self-efficacy. It also draws on Public and Patient Involvement contributors’ lived experiences and interpretations to enhance the relevance and depth of the findings.

Specific review questions included:

1) What is the effectiveness of online psychosocial interventions aimed at reducing caregiver burden?
2) What is the effectiveness of online psychosocial interventions aimed at improving self-efficacy?
3) What are the characteristics of online psychosocial interventions implemented to reduce caregiver burden and improve self-efficacy?
4) How can existing research evidence guide the refinement and future directions of online psychosocial interventions for informal caregivers of people with dementia?

In line with previous systematic reviews, we adopted a broad definition of psychosocial interventions. These interventions are understood as programs and initiatives targeting the psychological and social impacts of caregiving on daily life and long-term well-being (Gitlin et al., 2015). They have been acknowledged as drawing on a range of diverse mechanisms to support dementia caregiving, including professional support, psycho-education, behaviour management/skills training, counselling/psychotherapy, self-care/relaxation techniques, and environmental redesign to informal caregivers (Gitlin et al., 2015; Hoel et al., 2023; Wiegelmann et al., 2021). Typically involving multiple components, these interventions can be provided by such professionals as psychologists, social workers, and therapists, as well as lay and peer workers in online setting.

## 2 Methods

The review protocol was registered in PROSPERO (CRD42023392232).

### 2.1 Search strategy

The search strategy was developed by the primary author and supported by a librarian and incorporated the following six databases: MEDLINE (OVID), Embase (Elsevier), Web of Science, CINAHL (EBSCOhost), PsycINFO and Scopus. The search strategy used a combination of Medical Subject Headings and keywords for the following three distinct concepts: caregivers, dementia, and online intervention (Supplementary Material). The databases were searched for all publications available up to March 18, 2025. To identify additional articles, the reference lists of included articles were hand searched. Corresponding authors of the studies were contacted where either there was insufficient information to determine eligibility or to conduct meta-analysis

### 2.2 Inclusion and exclusion criteria

Table 1 presents the inclusion and exclusion criteria, which were set following the Population, Intervention, Comparator, Outcomes, and Study design domains.

**Table 1:** Inclusion and exclusion criteria.

**Study Characteristics**	**Inclusion Criteria**	**Exclusion Criteria**
**Participants**	• Adult (18 years old and over) informal caregivers (e.g., family member, friend) currently taking care of people with dementia. • There were no limitations on the type of dementia and disease duration of the caregiving recipients. • There were no limitations on the length of time caregivers are providing care for or the intensity/type of care provided. • Caregivers providing care for people with dementia diagnosed with other comorbidities (e.g., anxiety, depression, and diabetes) were included once the intervention is focused on dementia rather than any other comorbidity.	• Caregivers providing care for inpatient dementia patients. • Caregivers externally employed to provide care.
**Intervention**	• Any form of online psychosocial intervention to reduce caregiving burden or improve self-efficacy. In this review online psychosocial intervention are psychological and social interventions by providers including psychologists, psychiatrists, social workers, counsellors/therapists, primary care and other nonpsychiatric physicians, nurses, physical and occupational therapists, religious leaders, lay and peer providers, paraprofessionals and caregivers, and automated providers. • Community-based intervention. • Intervention without restrictions in relation to frequency, intensity	• The intervention contained only DVD programs or telephone/email support services. • The intervention involved face-to-face components. • The intervention focused solely on people with dementia.
**Comparators**	• Comparator groups must be informal caregivers of people with dementia. • Comparator groups receiving usual care or waitlist control. In this review, usual care refers to the standard or routine services and resources available to caregivers in their usual healthcare or community context, without additional structured interventions provided by the study. • Comparators without restrictions in relation to dose, intensity, or duration.	• Comparator group including caregivers for people with other neurological or non-neurological disorders. • Comparator groups receiving active or attention control interventions.
**Outcome measure**	• Caregiving burden measured by standardized scales (e.g., Zarit Burden Interview, the Burden Scale for Family Caregivers, the Burden Visual Analog scale) • Self-efficacy measured by standardized scales (e.g., Generalized Self-Efficacy Scale, Caregiver Self-Efficacy Scale)	• Outcomes reported are not related to caregiving burden or self-efficacy (e.g., depression, anxiety)
**Study design**	• Randomized controlled trials with any type of true random allocation method. • Studies published in peer review journals.	• Qualitative studies, observational studies, protocols, or systematic reviews. • Full-text articles are not available.

### 2.3 Study records and selection

The records identified from the search were uploaded to EndNote 20. Following the removal of duplicates, titles and abstracts of the identified records were exported to Rayyan to support the study selection process. Titles and abstracts were screened by two reviewers (QL and DZ) independently using the eligibility criteria. Subsequently, full texts of relevant articles were retrieved and screened by the two reviewers (QL and DZ) independently using the same eligibility criteria. A third reviewer (SH) was included to solve any disputes. Multiple reports of the same study were identified during the screening process to ensure that original studies and not reports are the units of interest for this review.

### 2.4 Data extraction

One reviewer (QL) extracted data from eligible studies with a structured data extraction worksheet capturing the key information from each study. To identify the Evidence for Equity, we also extracted the health equity information of the participants. In addition, a Public and Patient Involvement statement and any other information related to this form of design and consultation was also extracted. The extracted data were cross-checked and finalised with those obtained by another reviewer (DZ).

### 2.5 Quality appraisal

The Cochrane Risk of Bias Tool 2 was used to assess methods of included studies for risk of bias (Higgins et al., 2023). We conducted separate risk of bias assessments for burden and self-efficacy outcomes. Each included study was independently assessed for risk of bias by two reviewers from the author team (QL, EQ, and DZ). Disagreements were resolved by a third reviewer (SH). The key information for the assessment of risk of bias of each selected study was record and documented. The quality of the evidence was assessed using the Grades of Recommendation, Assessment, Development and Evaluation (GRADE) approach, a systematic and transparent framework for rating the certainty of evidence and strength of recommendations in health research. (Guyatt et al., 2011). It includes five assessment criteria: risk of bias, inconsistency of results, indirectness, imprecision, and publication bias. This system uses a baseline rating of high for randomized controlled trials, which can be downgraded to moderate, low, or very low, reflecting the general confidence in the pooled effect size.

### 2.5 Data synthesis

Meta-analysis was performed to examine the effectiveness of online psychosocial interventions for caregiver burden and self-efficacy by using RevMan 5.4. Considering that the outcomes were measured with different validated scales, a standardized mean difference (SMD) with a 95 % confidence interval (CI) was calculated to synthesize the pooled effects of the continuous data. A random effects model was used because of the anticipated heterogeneity across studies. A *p* value < 0.05 was considered statistically significant. Heterogeneity was assessed by conducting a χ^2^ test and using *I*^2^ statistic. A *p* value of 0.10 is used to determine statistical significance (Higgins et al., 2023). Thresholds for the interpretation of the *I*^2^ statistic are as follows: 0 % to 40 %: might not be important; 30 % to 60 %: may represent moderate heterogeneity; 50 % to 90 %: may represent substantial heterogeneity; 75 % to 100 %: considerable heterogeneity. For this meta-analysis, the short-term (immediately post-intervention) intervention effects were pooled and reported when applicable. Sensitivity analysis was conducted to assess whether the findings of this review were robust. We excluded studies with high risk of bias to explore the impact of risk of bias on the conclusions. Where study results could not be pooled statistically, we used a narrative analysis to investigate and report on the findings of the included studies.

### 2.7 Patient and public involvement

To address issues regarding the perceived relevance of systematic review findings and the concerns regarding the interpretation of those findings, a Public and Patient Involvement research panel with five contributors was established as a part of the systematic review protocol. In our review, the panel includes dementia caregivers and people living with dementia. Following the Cochrane review ecosystem and the Authors and Consumers Together Impacting on Evidence framework, contributors were involved in interpreting findings and writing and publishing review, with a level of involvement categorized as ‘contributing.’ (Pollock et al., 2019). They participated in online group meetings, supplemented by emails and phone calls. The sessions focused on exploring review findings, examining study characteristics, and discussing potential implications. Contributors also reviewed and provided feedback on the manuscript.

## 3 Results

The process of study search and selection is summarized in the Supplementary Material. A total of 17089 records were identified; after removing duplicates (*n =* 9373), reviews (*n =* 701) and protocols (*n =* 246), 6769 returns were retained and uploaded to Rayyan for screening. Finally, 19 eligible articles were included, and 13 of them (or their outcomes) were found suitable for meta-analysis.

### 3.1 Characteristics of included studies

Among the 19 studies included, eight were conducted in the United States of America (USA), two in the United Kingdom (UK), one each in Australia, Canada, France, Germany and Iran; four were multisite studies including one involving Australia and greater China, one involving Canada and the USA, one involving England, France, and Germany, and one involving the UK, Spain, and Greece. (Table 2)

**Table 2:** Characteristics of the included studies and participants.

**Authors**	**Country**	**Study design**	**Sample size of caregivers**	**Age (Year)**	**Sex**	**Relationship to patient**	**Caregiving duration**
Beauchamp et al., 2005	USA	RCT(2-arm)	IG: 150 CG: 149	**Mean (SD), range** 46.9 (12.2), 19.2-84.3	**Female, %** 73	**Parent, *n* ( %):** 67 (200) **Spouse or Partner, *n* ( %):** 7 (21) **Other relative, *n* ( %):** 23 (69) **Nonrelative, *n* ( %):** 3 (9)	Not reported
Blackberry et al., 2023	Australia	Stepped-wedge cluster RCT	*n* = 113 caregivers in 12 communities	**Mean (SD)** 60 (12)	**Female, *n* ( %)** 32 (86)	**Parent, *n* ( %):** 18 (48) **Spouse, *n* ( %):** 14 (38) **Sibling, *n* ( %):** 4 (10) **Friend, *n* ( %):** 1 (3)	**Years, *n* ( %)** <2: 13 (35) 2–6: 13 (35) >6: 11 (30)
Castillo et al., 2023	Canada, USA	RCT(3-arm)	IG1: 45 IG2: 44 CG: 41	**Mean (SD)** IG1: 54.62 (15.22) IG2: 55.09 (13.57) CG: 56.38 (11.34)	Not reported	Not reported	**Years, M (SD)** IG1: 3.78 (3.54) IG2: 3.07 (2.26) CG: 4.15 (3.42)
Cristancho-Lacroix et al., 2015	France	RCT(2-arm)	IG: 25 CG: 24	**Mean (SD)** IG: 64.2 (10.3) CG: 59.0 (12.4)	**Female, *n* ( %)** IG: 16 (64) CG: 16 (67)	**Children of persons with Alzheimer's disease, *n* ( %)** IG: 16 (64); CG: 13 (54)	Not reported
Duggleby et al., 2018	Canada	RCT(2-arm)	IG: 101 CG: 98	**Mean (SD)** IG: 63.4 (12.2) CG: 63.9 (11.1)	**Female, *n* ( %)** IG: 79 (78) CG: 82 (84)	**Spouse or life partner, *n* ( %)** IG: 51 (50); CG: 47 (48) **Son or daughter, *n* ( %)** IG: 47 (47); CG: 44 (45) **Other, *n* ( %)** IG: 3 (3); CG: 7 (7)	**Years, Mean (SD)** IG: 4.1 (3.8) CG: 4.6 (4.5)
Fowler et al., 2016	USA	RCT(2-arm)	IG: 15 CG: 13	**Mean (SD)** IG: 60 (12.77) CG: 67 (12.20)	**Female, *n* ( %)** IG: 4 (31) CG: 9 (69)	**Spouse, *n* ( %):** IG: 4 (27); CG: 9 (69) **Parent, *n* ( %):** IG: 6 (40); CG: 3 (23) **Mother-in-law, *n* ( %):** IG: 1 (6.7); CG: 1 (8) **Other, *n* ( %):** IG: 4 (27); CG: Not reported	**Months, M (SD)** IG: 57 (38.78) CG: 93 (83.41)
Gaugler, et al., 2023	USA	RCT(2-arm)	IG: 21 CG: 22	**Mean (SD)** 61.84 (12.89)	**Female, *n* ( %)** **IG:** 17 (39.5) **CG:** 15 (34.9)	**Spouse or partner, *n* ( %):**15 (34.9) **Daughter or son, *n* ( %):** 24 (55.8) **Daughter or son-in-law, *n* ( %):**2 (4.7) **Sister or brother, *n* ( %):**1 (2.3) **Other, *n* ( %):**1 (2.3)	Not reported
Gustafson et al., 2019	USA	RCT(2-arm)	IG: 16 CG: 15	*n***( %)** IG: 55-64: 3 (18.8), 65-74: 7 (43.8), 75+: 6 (37.5) CG: 55-64: 3 (20), 65-74: 9 (60), 75+: 3 (20)	**Female, *n* ( %)** IG: 11 (68.8) CG: 8 (53.3)	**Spouse or partner, *n* ( %)** IG: 15 (93.8); CG: 13 (86.7) **Adult child, *n* ( %)** IG: 0 (0); CG: 1 (6.7) **Other relative, *n* ( %)** IG: 1 (6.3); CG: 1 (6.7)	Not reported
Han et al., 2023	USA	RCT(2-arm)	IG: 9 CG: 10	**Mean (SD)** **IG:** 56 (20.1) **CG:** 53.2 (10.7)	**Female, *n* ( %)** **IG:** 9 (100) **CG:** 10 (100)	**Daughter, *n* ( %):** 15 (78.9) **Wife, *n* ( %):** 2 (10.5) **Sister, *n* ( %):** 1 (5.3) **Granddaughter, *n* ( %):** 1 (5.3)	**Years, M (SD)** IG: 2.9 (1.4) CG: 4.7 (2.8)
Hepburn et al., 2021	USA	RCT(3-arm), randomized 2:2:1 to active, attention control, and usual care arms	Active intervention group: 96 Attention control group: 111 Waitlist control group: 54	**Mean (SD)** Active intervention group: 66 (10.9) Attention control group: 63.8 (11.6) Waitlist control group: 63.7 (10.7)	**Female, *n* ( %)** Active intervention group: 72 (75) Attention control group: 73 (65.8) Waitlist control group: 39 (72.2)	**Spouse, *n* ( %)** Active intervention group: 69 (71.9) Attention control group: 68 (61.3) Waitlist control group: 35 (64.8)	Not reported
Kales et al., 2018	USA	RCT(2-arm)	IG: 27 CG: 30	**Mean (SD)** IG: 65.5 (11.8) CG: 66.2 (15.9)	**Male, *n* ( %)** IG: 8 (29) CG: 6 (19)	**Person with Dementia is Spouse, *n* ( %)** IG: 13 (48); CG: 15 (50)	Not reported
Meichsner et al., 2019	Germany	RCT(2-arm)	IG: 19 CG: 18	**Mean (SD), range** IG: 63.00 (9.40), 45-82 CG: 61.17 (10.14), 41-76	**Female, *n* ( %)** IG: 14 (73.7) CG: 15 (83.8)	**Spouse, *n* ( %)** IG: 15 (78.9); CG: 11 (61.1) **Adult child, *n* ( %)** IG: 4 (21.1); CG: 7 (38.9)	**Years, Mean (SD), range** IG: 4.16 (2.71), <1-10 CG: 4.94 (3.68), <1-15
Metcalfe et al., 2019	England, France, and Germany	RCT(2-arm)	IG: 30 CG: 31	**Mean (SD)** IG: 57.6 (10.5) CG: 57.2 (9.9)	**Female, %** IG: 60.0 CG: 61.3	Not reported	**Years, M (SD)** IG: 1.8 (1.1) CG: 2.6 (1.7)
Moskowitz et al., 2019	USA	RCT(2-arm)	IG: 86 CG: 84	**Mean (SD)** IG: 63.03 (9.36) CG: 62.77 (10.09)	**Female, *n* ( %)** IG: 73 (84.9) CG: 70 (83.3)	**Spouse/sig other, *n* ( %)** IG: 62 (72.1); CG: 52 (61.9) **Parent (Child?), %** IG: 23 (26.7); CG: 23 (27.4) **Other Family member, *n* ( %)** IG: 1 (1.2); CG: 7 (8.3) **Friend, *n* ( %)** IG: 0 (0); CG: 2 (2.4)	**Years, Mean (SD)** IG: 4.53 (4.31) CG: 3.89 (3.91)
Patel, et al., 2023	UK	RCT(2-arm)	IG: 15 CG: 24	**Mean (SD)** IG: 61.80 (8.79) CG: 59.79 (12.11)	**Female, *n* ( %)** IG: 12 (80) CG: 17 (70.83)	**Child, *n* ( %):** IG: 8 (53.33); CG: 9 (37.50) **Spouse, *n* ( %):** IG: 5 (33.33); CG: 9 (37.50) **Son-in-law/daughter-in-law, *n* ( %):** IG: 1 (6.67); CG: 0 (0) **Other relative, *n* ( %):** IG: 1 (6.67); CG: 6 (25)	Not reported
Salehinejad et al., 2022	Iran	RCT(2-arm)	IG: 25 CG: 25	**N ( %)** IG: ≤40: 7 (28); 41-50: 10 (40); ≥51: 8 (32) CG: ≤40: 9 (36): 41-50: 9 (36); ≥51: 7 (28)	**Female, *n* ( %)** IG: 15 (60) CG: 16 (64)	**Spouse, *n* ( %):** IG: 4 (16); CG: 5 (20) **Children, *n* ( %):** IG: 20 (80); CG: 14 (56) **Other, *n* ( %):** IG: 1 (4); CG: 6 (24)	**Years, n ( %)** IG: ≤1: 2 (8); 1-3: 14 (56); ≥3: 9 (36) CG: ≤1: 6 (24); 1-3: 12 (48); ≥3: 7 (28)
Torkamani et al., 2014	UK, Spain, Greece	Multi-center RCT(2-arm)	IG: 30 CG: 30	**Mean (SD)** IG: 57.57 (12.50) CG: 63.93 (14.74)	Not reported	Not reported	Not reported
Windle et al., 2025	UK	Multi-center RCT(2-arm)	IG: 175 CG: 177	**Mean (SD)** IG: 62.6 (12.0) CG: 61.9 (11.3)	**Female, *n* ( %)** IG: 140 (80) CG: 140 (79.1)	**Spouse/partner, *n* ( %):** IG: 77 (44.0); CG: 75 (42.4) **Sibling, *n* ( %):** IG: 2 (1.1); CG: 3 (1.7) **Child, *n* ( %):** IG: 87 (49.7); CG: 90 (50.9) **Parent, *n* ( %):** IG: 0 (0); CG: 0 (0) **Friend, *n* ( %):** IG: 1 (0.6); CG: 2 (1.1) **Other, *n* ( %):** IG: 8 (4.6); CG: 7 (4.0)	Not reported
Xiao et al., 2024	Australia & greater China	Multi-center RCT(2-arm)	IG: 131 CG: 135	**Mean (SD)** IG: 52.9 (15.1) CG: 53.0 (13.8)	**Female, *n* ( %)** IG: 104 (79.4) CG: 101 (74.8)	**Spouse, *n* ( %):** IG: 26 (19.8); CG: 27 (20.0) **Adult child carers, *n* ( %):** IG: 95 (72.5); CG: 101 (74.8) **Others, *n* ( %):** IG: 10 (7.6); CG: 7 (5.2)	**Duration on carer role (years): median (IQR)** IG: 3 (1–5) CG: 3 (2–5)

Note: CG: Control group; IG: Intervention group; IQR: Interquartile range; M: Mean; *n*: Number of participants; RCT: Randomized controlled trial; SD: Standard deviation; UK: United Kingdom; USA: United States of America.

### 3.2 Characteristics of the participants

The 19 studies included 2264 caregivers of people with various kinds of dementia, with sample size ranging from 19 to 352 participants (Table 2). Most caregivers were older adults, typically spouses or adult children of people with dementia. See Supplementary Material for additional information on health equity and care recipient characteristics.

### 3.3 Characteristics of interventions

The key characteristics of the interventions are summarized in Table 3, using items adapted from the Template for Intervention Description and Replication checklist (Hoffmann et al., 2014).

**Table 3:** Characteristics of the interventions in included studies.

**Authors**	**Intervention**						**Control**
	**Platform**	**Delivery mode**	**Degree of tailoring**	**Duration**	**Frequency**	**Intervenors**	
Beauchamp et al., 2005	Website: Caregiver's Friend: Dealing With Dementia	Individual	Tailored. Personalized content through a questionnaire based on caregiver situation and dementia severity.	30 Days	Self-paced	NA	Waitlist control
Blackberry et al., 2023	Website and mobile app (Verily Connect) Web-based videoconference	Blended: individual & group-based	Partially tailored. Local directories and peer discussions adapted to each community’s context.	32 weeks	Verily Connect: self-paced Videoconference: monthly	Peer groups in the web-based videoconference	Delayed intervention (stepped wedge)
Castillo et al., 2023	App 1: Dementia Talk App 2: CLEAR Dementia Care	Individual	Partially tailored. Users select behaviours and management strategies relevant to their caregiving situation.	Two weeks	Self-paced	NA	baseline, post-intervention and follow-up period measurement
Cristancho-Lacroix et al., 2015	Web-based fully automated psychoeducational program (called Diapason)	Individual	Standardized.	3 months	Weekly	A clinical psychologist to take part in discussion if necessary	Waitlist control
Duggleby et al., 2018	Website: My Tools 4 Care (MT4C)	Individual	Partially tailored. Personal reflection sections allow individual input.	3 months	Self-paced	NA	Participants in the educational control group (usual care) received a copy of the Alzheimer’s Society’s The Progression of Alzheimer’s Disease booklet, via email, after the first interview.
Fowler et al., 2016	Website: Virtual Healthcare Neighbourhood (VHN)	Individual	Partially tailored. Standardized content with individual interaction via “Ask the Expert.”	16 weeks	Weekly	Caregivers can have a one-on-one discussion with investigators in the “Ask the Expert” blog.	Completed the same study questionnaires and informed consent, used the VHN website solely to upload their sleep actigraphy information.
Gaugler, et al., 2023	Online tool (Care to Plan [CtP])	Individual	Tailored. Personalized support recommendations and local resources generated from individual caregiver assessments.	6 months	Self-paced		Usual care
Gustafson et al., 2019	Website: Dementia-CHESS (Dementia–Comprehensive Health Enhancement Support System)	Individual	Tailored. Interactive tools and specialist feedback provide individualized support and adaptive recommendations.	6 months	Self-paced	NA	The control group received a limited intervention consisting of a book for family caregivers of dementia patients, The 36-Hour Day. The book was mailed to the control group, and no other training was provided.
Han et al., 2023	Synchronous Zoom-based video conferences	Individual	Partially tailored. Counsellor adapted ACT sessions to each caregiver’s needs.	8 weeks	Weekly	A licensed professional counsellor with substantial clinical experiences and expertise in ACT	Participants in the control group maintained care as usual and received the same psychoeducation materials given to the participants in the ACT group during the study period. After the study period ended, the control group received links to online ACT resources.
Hepburn et al., 2021	Synchronous Zoom-based video conferences: Tele-Savvy	Group-based	Standardized. Structured group program.	43 days	Weekly	Expert facilitators with prior experience leading the in-person Savvy program	Waitlist control
Kales et al., 2018	Web based tool (called the WeCareAdvisor)	Individual	Tailored. DICE algorithm provides individualized strategies based on caregiver input.	One month	Self-paced	NA	Waitlist control
Meichsner et al., 2019	Website: the Tele.TAnDem.online blog	Individual	Tailored. Therapist adapted CBT modules to each participant’s main problems.	8 weeks	Weekly	Clinical psychologists	Waitlist control
Metcalfe et al., 2019	Web based program	Individual	Standardized.	6 weeks	Self-paced	NA	Waitlist control
Moskowitz et al., 2019	Synchronous WebEx meeting: LEAF (Life Enhancing Activities for Family caregivers)	Individual	Partially tailored. Final session personalized plans for continued skill practice.	6 weeks	Weekly	Trained facilitators	Waitlist control
Patel, et al., 2023	Synchronous videoconference	Group-based	Standardized.	4 hours	One-off	One trainee clinical psychologist	Usual care
Salehinejad et al., 2022	Website	Individual	Standardized.	2 months	Weekly	NA	Participants in the control group receive usual information, such as a neurologist’s semi-annual follow-up appointment, during which the family caregivers obtain illness information from the neurologist.
Torkamani et al., 2014	Computerized platform: ALADDIN (A technology pLatform for the Assisted living of Dementia elDerly INdividuals and their carers)	Individual	Partially tailored. Users could request contact and submit health updates.	6 months	Self-paced	NA	The participants in the control group were only assessed at the three time points, without any further contact or intervention.
Windle et al., 2025	A web-based system: iSupport	Individual	Standardized.	6 months	Self-paced	NA	The usual care group were sent a booklet about being a dementia carer, either as a hard copy or PDF and were provided access to the WHO version of iSupport at the end of the study.
Xiao et al., 2024	A web-based system: iSupport Carer peer support group: Synchronous WeChat/ WhatsApp meeting	Blended: individual & group-based	Partially tailored. Facilitators provided need-based access to information and support alongside standardized modules.	6 months	iSupport: self-paced peer support meeting: monthly	Peer support meeting: registered nurses, social workers and allied health professional.	Usual care

Note: ACT: Acceptance and Commitment Therapy; CBT: Cognitive Behavioural Therapy; DICE: Describe, Investigate, Create, Evaluate; iSupport: WHO iSupport for Dementia; NA: Not applicable; WHO: World Health Organization.

#### 3.3.1 Platforms, delivery modes and formats

The interventions were administered through various online platforms, including web-based programs, mobile applications, and videoconferencing tools. Online interventions typically follow two delivery modes—synchronous and asynchronous. Synchronous interventions involve real-time interaction among participants or facilitators (e.g., through videoconferencing), whereas asynchronous interventions allow participants to access materials and complete activities at their own pace (e.g., through websites). Most researchers employed asynchronous interventions via web-based platforms. In one study, researchers provided both website and mobile application versions of the asynchronous program along with synchronous videoconferencing (Blackberry et al., 2023). Four used synchronous videoconferences (Han et al., 2023; Hepburn et al., 2021; Moskowitz et al., 2019; Patel et al., 2023), and one combined a synchronous component with a website-based program (Xiao et al., 2024). One study used a mobile app alone (Castillo et al., 2023). In terms of delivery formats, online interventions were either individual or group-based. All website- and application-based interventions were delivered individually. Interventions that integrated asynchronous (website or applications) and synchronous videoconferencing components were delivered in a blended format, combining both individual and group-based approaches. Synchronous videoconference-only interventions were primarily group-based, except for Moskowitz et al. (2019), who provided individual sessions with trained facilitators.

#### 3.3.2 Frequency and duration

The total duration of interventions among included studies ranged from a single 4-hour session to 8 months. For most interventions delivered via websites and applications, the frequency was self-paced, and participants were encouraged to access the program at their convenience (Castillo et al., 2023; Duggleby et al., 2018; Gaugler et al., 2023; Gustafson et al., 2019; Kales et al., 2018; Metcalfe et al., 2019; Torkamani et al., 2014; Windle et al., 2025). In seven studies, weekly sessions were adopted throughout the intervention duration (Cristancho-Lacroix et al., 2015; Fowler et al., 2016; Han et al., 2023; Hepburn et al., 2021; Meichsner et al., 2019; Moskowitz et al., 2019; Salehinejad et al., 2022). In the two studies that integrated both asynchronous and synchronous components, the asynchronous part was self-paced, while the synchronous videoconference sessions took place monthly (Blackberry et al., 2023; Xiao et al., 2024).

#### 3.3.3 Content of the interventions

The content of psychosocial interventions varied among the 19 studies. Except for two studies that solely targeted caregiver self-care through positive emotion regulation skills (Moskowitz et al., 2019) and acceptance and commitment therapy (Han et al., 2023), all others were multi-component interventions. The main intervention components were information provision and education, caregiving tasks support, and caregiver self-care (Table 4).

**Table 4:** Contents of psychosocial interventions in included studies.

**Authors**	Information provision and education		Caregiving tasks support			Caregiver self-care		
	Dementia understanding and treatment	Care management	Decision- making guidance	Problem-solving techniques	Behavioral management	Psychological support	Social support	Personal growth guidance
Beauchamp et al., 2005	√	√		√	√	√	√	
Blackberry et al., 2023	√	√	√^#^	√^#^	√^#^		√	
Castillo et al., 2023**-app 1**	√	√		√^#^	√	√	√	
Castillo et al., 2023**-app 2**	√	√^#^			√^#^	√^#^		
Cristancho-Lacroix et al., 2015	√	√		√	√	√	√	
Duggleby et al., 2018	√^#^	√^#^				√		√
Fowler et al., 2016	√^#^	√^#^		√^#^	√^#^		√	
Gaugler, et al., 2023	√	√		√^#^	√^#^	√	√	
Gustafson et al., 2019	√	√	√	√	√	√	√	
Han et al., 2023						√	√	√
Hepburn et al., 2021	√	√	√	√	√	√	√^#^	√
Kales et al., 2018	√	√		√	√^#^		√	
Meichsner et al., 2019	√	√		√	√	√	√	√
Metcalfe et al., 2019	√	√		√	√	√^#^	√^#^	√^#^
Moskowitz et al., 2019						√		√
Patel, et al., 2023	√	√		√^#^	√^#^		√	
Salehinejad et al., 2022	√	√		√	√	√	√	√^#^
Torkamani et al., 2014	√	√		√^#^			√	
Windle et al., 2025	√	√	√	√	√	√	√	√
Xiao et al., 2024	√	√	√	√	√	√	√	√

Note: Castillo et al. (2023) included two intervention groups using two different apps; these are presented separately as app 1 and app 2 to reflect their distinct intervention content. “#” indicates uncertainty due to a lack of clarity in the source material; while the element is likely included, the information provided is not detailed enough to confirm it.

Information provision and education formed a significant part of these interventions, including informational materials on the dementia condition and its treatment, as well as guidance on care management. Caregiving tasks support involved decision-making guidance, problem-solving techniques, and behavioural management skills. For example, a ‘tools area’ section on a website provided a step-by-step decision guide to help caregivers clarify situations and needs when considering placing a family member in specialized care (Gustafson et al., 2019). Others incorporated functions in applications allowing users to monitor the frequency and intensity of observed behaviours in individuals with dementia and select appropriate management strategies (Castillo et al., 2023). Caregiver self-care encouraged caregivers to reflect on their own self-care needs, offering social and psychological support (e.g., stress management), as well as opportunities for personal growth. This included a peer support group (Blackberry et al., 2023; Xiao et al., 2024) and networking component on the website (e.g., online discussion group/forum) (Cristancho-Lacroix et al., 2015; Salehinejad et al., 2022; Torkamani et al., 2014), enabling caregivers to communicate with each other. Additionally, certain programs encouraged caregivers to explore their own interests and develop a repertoire of activities to engage in when they had the opportunity, emphasizing personal growth (Duggleby et al., 2018; Han et al., 2023; Hepburn et al., 2021; Meichsner et al., 2019; Moskowitz et al., 2019; Windle et al., 2025; Xiao et al., 2024). The degree of tailoring also varied across interventions, ranging from standardized content to fully individualized programs that generated personalized strategies based on caregiver input (Table 3). (See detailed intervention content in Supplementary Material).

### 3.4 Characteristics of the comparators

In seven studies, the caregivers in the control group were on a waitlist and received the online psychosocial intervention after the intervention period (Beauchamp et al., 2005; Cristancho-Lacroix et al., 2015; Hepburn et al., 2021; Kales et al., 2018; Meichsner et al., 2019; Metcalfe et al., 2019; Moskowitz et al., 2019). In the remaining studies, comparator groups followed a *“*usual care “protocol, meaning that participants continued to access the standard or routine services available in their healthcare or community settings, without any additional structured interventions. For example, in Salehinejad et al. (2022), participants in the control group received standard information, such as a neurologist’s semi-annual follow-up appointment. In Duggleby et al. (2018) and Gustafson et al. (2019), participants were provided with a book or booklet, reflecting resources typically accessible to caregivers. In Fowler et al. (2016) and Torkamani et al. (2014), participants completed outcome assessments only during the study period. In Castillo et al. (2023), participants were asked to report information on care management.

### 3.5 Caregivers’ outcomes and measurements

In this review, the outcomes of interest were caregiver burden and self-efficacy. The outcome measurement timepoints of included studies were baseline, post-intervention and follow-up. In the included studies, apart from burden and self-efficacy, additional outcomes of interest were reported in individual studies. Table 5 summarises the outcome variables and measurements used across all the 19 studies at different measurement timepoints.

**Table 5:** Outcomes and measurements in included studies.

**Authors**	**Measures for burden/self-efficacy**	**Follow-up duration**	**Findings of burden/self-efficacy**	**Findings of other outcomes of interests**
Beauchamp et al., 2005	Six self-efficacy questions regarding areas of caregiving that were emphasized in the intervention. Participants responded on a 7- point Likert scale from 1 (not at all confident) to 7 (extremely confident; a=0.85).	NA	Self-efficacy ↑post intervention	• Intention to get support, caregiver gain ↑post intervention; caregiver stress, caregiver strain, depression and anxiety ↓post intervention. • No significant between-group difference was found in the frequency of employing coping skills post intervention.
Blackberry et al., 2023	Zarit Burden Interview (ZBI)	5 months	No significant difference was found in burden from baseline to the end of the implementation period.	• Caregiver social support ↑post intervention. (follow-up results were limited by available data)
Castillo et al., 2023	Zarit caregiver burden interview (ZBI)	3 weeks	No significant between-group difference was found in burden post intervention and follow-up.	• No significant between-group difference was found in knowledge about dementia and perceived stress post intervention and in follow-up.
Cristancho-Lacroix et al., 2015	Revised Scale for Caregiving Self-Efficacy (RSCS) Zarit Burden Interview	3 months	No significant between-group difference was found in burden and self-efficacy post intervention and follow-up.	• Knowledge of the disease ↑post intervention, but no between-group difference was found follow-up. • No significant between-group difference was found in self-perceived stress, perception and reaction to cognitive or behavioral symptoms of persons with Alzheimer’s disease, depression, self-perceived heath, overall stress, and the caregivers- persons with Alzheimer’s disease relationship quality post intervention and follow-up.
Duggleby et al., 2018	General Self-Efficacy Scale (GSES)	3 months	No significant between-group difference was found in self-efficacy post intervention and follow-up.	• Herth Hope Index factor 2 scores ↑post intervention; no significant between-group difference was found in Herth Hope Index total score post intervention and follow-up. • No significant between-group difference was found in health-related quality of life post intervention and follow-up.
Fowler et al., 2016	General Self-Efficacy Scale	NA	No significant between-group difference was found in overall scores of self-efficacy; Scores on specific items on the General Self-Efficacy Scale ↑ post intervention.	• No significant between-group difference was found in insomnia severity and sleep actigraphy post intervention.
Gaugler, et al., 2023	• Zarit Burden Interview (ZBI) • Fortinsky Dementia Caregiver Self-Efficacy Scale (unofficial)	NA	No significant between-group difference was found in burden and self-efficacy post intervention	• No significant between-group difference was found in caregiver stress and depressive symptoms post intervention.
Gustafson et al., 2019	Caregiver load scale	NA	No significant between-group difference was found in burden post intervention.	• No significant between-group difference was found in family conflict, satisfaction with care decisions, social support, loneliness, anxiety, depression and coping competence post intervention.
Han et al., 2023	Zarit Burden Interview (ZBI)	1 month	No significant between-group difference was found in burden post intervention and follow up.	• Reduction in grief ↑post intervention but no between-group difference was found follow-up. • No significant between-group difference was found in depressive symptoms, anxiety, and stress, feelings of guilt in caregivers, psychological QoL, psychological flexibility, cognitive fusion, engagement in meaningful activities, self-compassion post intervention and follow-up.
Hepburn et al., 2021	The Zarit Burden Inventory	About 5 months	No significant between-group difference was found in burden post intervention and follow-up.	• Perceived stress, depression ↓post intervention and follow-up; caregiver mastery and reaction to care recipients’ behaviours ↑post intervention and follow-up. • No significant between-group difference was found in anxiety post intervention and follow-up.
Kales et al., 2018	12-item Zarit burden scale	NA	No significant between-group difference was found in burden post intervention.	• Caregiver distress ↓post intervention. • No significant between-group difference was found in caregiver confidence, caregiver stress, depression, negative communication, relationship closeness post intervention.
Meichsner et al., 2019	Burden of care was measured with a visual analog scale ranging from 0 = I do not feel burdened to 100 = I feel highly burdened	3 months	No significant between-group difference was found in burden post intervention and follow-up.	• No significant between-group difference was found in symptoms of depression post intervention. However, there was a significant interaction between T2 (follow-up) and study group. • Coping with anticipated loss (Absolute Loss) ↑post intervention but was not sustained at follow-up. • Utilization of well-being-related resources ↑ post intervention but was not sustained at follow-up. • No significant between-group differences were found in coping with predeath grief, resources related to daily hassles, and social support post intervention and follow-up.
Metcalfe et al., 2019	Revised Scale for Care‐giving Self‐Efficacy (RSCSE) Burden Scale for Family Caregivers (BSFC)	6 weeks	No significant between-group difference was found in burden and self-efficacy post intervention. (follow-up results were not reported)	• Caregiver perceived stress, caregivers' negative reactions to memory symptoms ↓post intervention. • No significant between-group difference was found in health-related quality of life post intervention. • (follow-up results were not reported)
Moskowitz et al., 2019	Zarit Burden Interview	6 months	No significant between-group difference was found in burden post intervention. (follow-up results were not reported)	• Positive emotion, physical health and positive aspects of caregiving ↑post intervention; depression and anxiety ↓post intervention. • No significant between-group difference was found in perceived stress, strain, negative emotion post intervention.
Patel, et al., 2023	Short-Form Zarit Burden Interview (ZBI-12)	1 month	No significant between-group difference was found in burden post intervention.	• No significant between-group difference was found in attitudes towards people with dementia, quality of the caregiver patient relationship, sense of competence, positive aspects of caregiving post intervention.
Salehinejad et al., 2022	Zarit Burden Interview	NA	Burden ↓post intervention	• Alzheimer disease knowledge and positive attitude towards the disease ↑post intervention.
Torkamani et al., 2014	Zarit Burden Interview	NA	No significant between-group difference was found in burden post intervention	• Quality of life ↑post intervention. • No significant between-group difference was found in distress and depression post intervention.
Windle et al., 2025	12-item Zarit burden Interview (ZBI-12)	NA	No significant between-group difference was found in burden post intervention	• No significant between-group difference was found in depression, resilience, dementia knowledge, quality of the care relationship, anxiety post intervention.
Xiao et al., 2024	Revised Scale for Caregiving Self-efficacy (RSCS)	3 months	Self-efficacy in controlling upsetting thoughts ↑post intervention, but no between-group difference was found follow-up.	• Mental-health-related quality of life ↑post intervention, but no between-group difference was found follow-up. • Distress reactions to changed behaviours ↓post intervention, but no between-group difference was found follow-up. • No significant between-group difference was found in social support, Quality of Life in Alzheimer's Disease post intervention and follow-up.

Note: This table summarizes outcome measures for caregivers only; NA: Not available, as the studies did not include follow-up assessments; ↑: Increase; ↓: Decrease.

### 3.6 Quality appraisal

#### 3.6.1 Burden

Supplementary Material, File 2 summarises the risk of bias assessment for studies on burden. Overall, six studies were assessed to have a high risk of bias, and nine had some concerns. Over half of the included studies (*n =* 8) had low risk of bias in the domain of “randomization process”, and the seven remaining studies were at some concerns. The high risk of bias was mainly identified in two domains: “deviations from intended interventions” (*n =* 3), due to insufficient information about deviations arising from the trial context and the absence of appropriate analyses estimating the effect of assignment to the intervention; and “missing outcome data” (*n =* 4), due to a lack of reported analysis methods that could correct for bias or demonstrate that the results were minimally affected. In the domain of “measurement of the outcome”, most studies were assessed as having some concerns of risk of bias (*n =* 13), while two were rated as high risk due to the use of less-validated assessment tools. In terms of “selection of the reported result”, one study incurred a high risk of bias because of not reporting results for a specified follow-up timepoint as outlined in the analysis plan.

#### 3.6.2 Self-efficacy

Supplementary Material, File 3 summaries the risk of bias assessment for studies on self-efficacy. Five studies had a high overall risk of bias, and two had some concerns. The primary source of high bias risk was detected in the “missing outcome data” domain across five studies, and additionally, in the domain of “deviations from the intended interventions” in three studies. No high risk of bias arose in the domain of the randomization process.

#### 3.6.3 GRADE assessment

When comparing online psychosocial interventions to usual care on reducing burden and improve self-efficacy, the GRADE assessment indicated that there was a low level of evidence certainty in the effect estimates in both outcomes. The level of evidence was downgraded primarily due to concerns related to the risk of bias, inconsistency, and imprecision (Supplementary Material).

### 3.7 Effectiveness of online psychosocial interventions for caregivers of people with dementia

The effects of the individual online psychosocial interventions post-intervention and in follow-up are presented in Table 5. Due to the lack of follow-up in over half of the included studies and variations in the duration of follow-up across the studies, we solely synthesized effects observed immediately after the intervention.

#### 3.7.1 Burden

Caregiver burden was set as an outcome measure in 15 studies; 13 were included in the meta-analysis. The pooled results showed no statistically-significant effect of online psychosocial interventions on reducing caregiver burden (SMD: −0.06, 95 % CI: −0.25, 0.12, *p* =0.50). (Supplementary Material) Moderate statistical heterogeneity was observed for the burden outcome (*I*² = 47 %). In the sensitivity analysis, studies with a high risk of bias were excluded, and the results remained consistent. Although attempts were made to contact the original investigators, two studies did not have sufficient data (means, standard deviations or sample size) to be included for meta-analysis (Kales et al., 2018; Metcalfe et al., 2019). In a multi-site randomized controlled pilot study, an online program for young-onset dementia caregivers showed no statistically-significant post-intervention burden score differences (Metcalfe et al., 2019). Researchers who conducted another included study on a web-based tool found no statistically-significant between-group differences in burden change scores after a 1-month intervention (Kales et al., 2018).

#### 3.7.2 Self-efficacy

Seven studies reported the effect of online intervention on self-efficacy. The meta-analysis of available studies (total *n =* 530 participants) indicated statistically-significant post-intervention effect on caregivers’ self-efficacy [SMD: 0.20, 95 % CI: (0.03, 0.37), *p* =0.02] (Supplementary Material), with a small effect size and a very low heterogeneity (*I*^2^ = 0 %). For studies that were not eligible for meta-analysis, three investigated the effects of web-based interventions on enhancing self-efficacy across three domains, and reported the outcomes accordingly. Two of these studies did not show a statistically-significant difference between groups within any of the domains after intervention (Cristancho-Lacroix et al., 2015; Metcalfe et al., 2019), while one study indicated significant improvement in the domain of ‘controlling upsetting thoughts’ (Xiao et al., 2024). Another included study assessed caregivers’ self-efficacy based on eight different items, with no statistically-significant between-group differences in overall scores before or after intervention (*p* > 0.05), However, statistically-significant differences were noted in specific items on the General Self-Efficacy Scale, with the intervention group scoring higher on ‘Compared to other people, I can do most tasks very well,’ and ‘When facing difficult tasks, I am certain that I will accomplish them’ after intervention (Fowler et al., 2016), suggesting positive effects on these items.

### 3.8 Reports on patient and public involvement

In the 19 included studies, Public and Patient Involvement was reported in two studies. Windle et al. 2025 reported the involvement of people living with dementia and their carers in the development of their study (Windle et al., 2022). Another study acknowledged the contribution by thanking an experienced and skilful caregiver for his advice, support, and encouragement throughout the study (Salehinejad et al., 2022).

## 4 Discussion

In this systematic review, we aimed to provide a rigorous synthesis of evidence on online psychosocial interventions for informal caregivers of people with dementia, with a focus on burden and self-efficacy. Based on our findings, we have contributed to the scientific understanding of existing evidence for both outcomes.

We found that existing online psychosocial interventions had no effect on reducing caregiver burden. This contrasts with the findings of Deeken et al. (2019), who reported a small but significant post-intervention effect on caregiver burden after evaluating a wide range of technology-based interventions (telephone; DVD/video; internet-base; combined approaches) with broad control conditions, such as usual care and standard control. However, Leng et al. (2020), who focused only on telephone- and internet-based interventions with consistent control conditions, found no significant improvements in caregiver burden – aligning with our findings. When considering the broader landscape of non-pharmacological interventions for dementia caregivers, Cheng and Zhang (2020) found, in a comprehensive meta-review, only a weak effect on caregiver burden, regardless of the modality (e.g. face-to-face vs. technology-based). They also revealed that technology-based interventions showed greater inconsistency in their outcomes compared to other types of interventions. While it may have been assumed that this inconsistency was due to variations in delivery modes, study designs and intervention focuses, we illustrated that similar inconsistencies in results exist when focusing only on randomized controlled trials, informal caregivers, and online psychosocial interventions.

Nevertheless, our synthesis findings on burden should be interpreted with caution. We assessed the overall certainty of the evidence as low, primarily due to concerns about risk of bias, inconsistency, and imprecision. We found several studies had methodological limitations, and moderate heterogeneity was observed across trials, with variations in effect direction and limited overlap in CIs. In addition, the pooled effect estimate was imprecise, with a CI that included both potential benefit and no effect, limiting confidence in the true magnitude of the intervention’s impact. Also, we focused on ensuring the quality of evidence by including only randomized controlled trials, but it is noteworthy that among these studies, nearly one-third were pilot studies. This could also contribute to the null results, as pilot studies are less capable of testing for statistical significance between groups due to their small populations, exploratory nature, and emphasis on feasibility and refinement (Leon et al., 2011). Caregiving burden is widely recognized as a key factor affecting the psychological well-being of family caregivers (Bongelli et al., 2024; Tu et al., 2022). Targeting specific sources of burden may help prevent adverse outcomes for both caregivers and people with dementia (Kuzuya et al., 2011; Tu et al., 2022). This has prompted calls for interventions that reduce burden and strengthen support (Encinas-Monge et al., 2024). Researchers should conduct methodologically rigorous trials to evaluate the effectiveness of online psychosocial interventions in reducing caregiver burden.

In terms of self-efficacy, we identified seven studies that assessed the impacts of online psychosocial interventions on it. The pooled results demonstrated an improvement in self-efficacy after intervention. Our finding is consistent with a previous systematic review and meta-analysis exploring telephone and internet-based supportive interventions for family caregivers of people with dementia (Leng et al., 2020). Given the risk of bias in the included studies, the overall certainty of the evidence is also low. Based on prior studies that suggested an association among self-efficacy, reduced burden, and improved coping with behavioural issues in Alzheimer's disease caregivers, we intended to investigate the potential impact of online psychosocial interventions on self-efficacy and whether this, in turn, might affect the burden outcomes. Unfortunately, due to the limited number of studies available, subgroup analysis could not be conducted. Among included studies, three studies specifically addressed both burden and self-efficacy (Cristancho-Lacroix et al., 2015; Gaugler et al., 2023; Metcalfe et al., 2019). However, in these three studies, no significant effects were observed on either outcome. Self-efficacy plays an important role in caregiver outcomes (Wilks et al., 2025). Considering its mediator role between caregiver variables (i.e., preparedness, income, and social networks) and health outcomes, it is suggested that strategies to improve caregiver self-efficacy should be an integral component of caregiver interventions (Phongtankuel et al., 2023). With only seven studies identified in the review that included self-efficacy as an outcome, we have also highlighted a lack of emphasis on positive outcomes in current intervention research. Researchers on dementia caregiving have largely focused on negative outcomes, such as depression and anxiety; however, emphasizing positive aspects like self-efficacy is crucial for a holistic understanding of caregiver experiences (Quinn, Gamble, et al., 2024; Quinn et al., 2019). Caregivers who identify beneficial aspects of their role experience less burden and improved health outcomes (Wang et al., 2022; Yuan et al., 2023). Acknowledging these benefits not only helps in developing targeted interventions to support caregivers but also promotes a more balanced view that recognizes the dual nature of caregiving (Quinn, Toms, et al., 2024).

### 4.1 Patient and public involvement: perspectives on findings

To further ground the findings of this review in real-world experiences, insights from the public panel were incorporated to deepen our interpretation. Drawing on their experiences as dementia caregivers and individuals living with dementia, the panel highlighted several key gaps of online psychosocial interventions that need to be addressed. In terms of intervention design, they noted the duration of interventions might explain the observed results, with most interventions in the included studies lasting no longer than 3 months. Contributors felt online interventions might require a longer duration for effective engagement, and, while short-term interventions can facilitate relationship-building among participants, achieving the intended impact within such a brief duration might prove challenging (Rozental et al., 2015).

In terms of the nature of intervention delivery, contributors highlighted the significant isolation experienced by caregivers (Azevedo et al., 2021), suggesting that group-based interventions could be more effective in addressing their needs. Such a setting addresses the challenges of isolation and self-doubt by providing a platform for caregivers to exchange ideas and share experiences (Daynes-Kearney & Gallagher, 2023). The emphasis lies not in offering advice, recognizing the uniqueness of each situation, but rather in fostering a supportive environment where caregivers can share personal experiences, hopes, and strengths (Daynes-Kearney & Gallagher, 2025).

Regarding the intervention components, while providing educational information about dementia and caregiving skills is essential, contributors emphasized the critical need for caregivers to receive techniques and approaches to identify valuable resources and support, which is not sufficiently emphasized in the studies included in the review. This suggestion has also been noted in the international literature (Soong et al., 2020). Additionally, caregivers exhibit an increased need for self-care support throughout all stages of caregiving, especially as the care recipient’s condition progresses and requires more intensive care (Lin et al., 2023), which was less emphasized in the included studies. Addressing these gaps by incorporating practical resource identification and ongoing self-care strategies into interventions could enhance their relevance and effectiveness for caregivers.

### 4.2 Directions and areas for future work

Taking together, the findings of this review and the Public and Patient Involvement perspectives, we argue that there are two key areas of development that should be considered for the future directions of work in this field.

The first area of development is with respect to design of online interventions. Given the low certainty of the evidence for both outcomes, we were unable to identify key features with certainty that will consistently enhance effects. Nevertheless, there are a number of considerations worth noting regarding the features of current studies, which potentially reflect some positive value when attempting to address the targeted outcomes. These considerations are now briefly described, with the aid of the broader scientific literature on intervention design.

A central consideration in designing future interventions is the importance of clearly focusing on key outcomes. Many studies in our review assessed burden and self-efficacy as secondary outcomes, while their primary focus was directed toward other factors, such as caregiver distress (Kales et al., 2018), perceived stress (Cristancho-Lacroix et al., 2015; Hepburn et al., 2021), depression (Meichsner et al., 2019), and positive emotion (Moskowitz et al., 2019). As secondary outcomes, burden and self-efficacy are unlikely to have served as the key consideration within the intervention design, potentially limiting the impact of any intervention on these outcomes. Caregiver burden and self-efficacy have been reported to be closely associated with caregivers’ physical and psychological well-being (Bongelli et al., 2024; Phongtankuel et al., 2023), which in turn can significantly affect the well-being and quality of care experienced by the care recipient (Kuzuya et al., 2011). Ensuring that interventions are explicitly designed around these core outcomes may enhance their effectiveness and relevance, particularly when addressing the complex and evolving needs of dementia caregivers.

Nearly all the included studies employed a multi-component content intervention design. These components can be broadly categorized into three aspects: information provision and education, caregiving tasks support, and caregiver self-care (Table 4). While the multi-component interventions may seem diverse, they share core elements of psychosocial interventions that address caregivers’ needs for psychological and social support. Previous researchers have suggested that, compared to single-intervention approaches (such as education/skills-based interventions alone), multi-component interventions that combine two or more conceptually distinct approaches are likely to be more effective in reducing caregiver burden (Williams et al., 2019).

Most interventions were delivered asynchronously through self-guided websites or applications. This approach may enhance accessibility and autonomy by allowing caregivers to engage with materials at their own pace and revisit content when needed. Such flexibility can promote self-reflection and self-directed learning, which may contribute to improved coping and self-efficacy (Aung et al., 2025; Grapp et al., 2022). However, the accessibility and engagement of these interventions is uncertain due to the asynchronous nature. Although caregivers can engage with the content at their own pace based on self-determined needs, this flexibility can lead to inconsistent engagement, with some struggling with motivation or time management. In contrast, interventions with a synchronous or interactive nature may foster greater motivation, accountability, and social connection, which can enhance emotional support and sustained participation (Ayoub et al., 2022). The lack of immediate feedback in asynchronous formats may also result in misunderstandings or misinterpretations of the information, potentially diminishing the overall effectiveness of the intervention for users. Given the diversity of delivery modes, it remains difficult to determine which approach is most favourable, and researchers should explore how different formats influence engagement and outcomes.

The final consideration in intervention design relates to the format—whether it is delivered individually or in a group-based format. Dickinson et al. (2017), in an umbrella review of systematic reviews, noted that while well-designed, clearly-structured multi-component interventions can help maintain the psychological health of caregivers, delivering them through a support group format may further enhance effectiveness (Dickinson et al., 2017). This may become particularly important in the context of online interventions, where real-time interaction may be lacking. The benefits of group-based intervention include the efficiency of support, reducing social isolation, peer support, and providing opportunities to share experiences and emotions (McLoughlin, 2022). However, the use of a group-based approach was relatively uncommon among the included studies. This highlights the underutilization of group-based online psychosocial interventions in practice. It is also important to recognise that caregivers differ in their preferences for individual versus group formats. While some may value the social connection of a group, others may feel less comfortable or prefer a more private and self-paced experience. These variations emphasise the importance of incorporating lived experience in developing interventions that are flexible and responsive to individual needs. Overall, group-based formats show potential for improving online interventions, their consistent effectiveness requires further investigation.

Notably, several post-COVID-19 studies included in this review already reflect a move toward more flexible and interactive formats. These recent interventions incorporated self-paced web systems with peer or professional support, videoconference-based sessions, and hybrid or application-based approaches (e.g., Xiao et al., 2024; Han et al., 2023; Blackberry et al., 2023). This trend suggests that online psychosocial interventions are evolving toward greater accessibility and social connection, directions that future work could further develop and evaluate.

The second area of development is with respect to methodological aspects of intervention assessments and the quality of evidence provided. Each of these elements may have influenced the outcomes reported in the included studies. One priority is reducing the risk of bias, particularly by minimizing deviations from intended interventions, enhancing transparency in analysis plans, and using more sophisticated measurement tools that better capture the complex and multidimensional nature of caregiver burden and self-efficacy. Studies should also adopt appropriate sample sizes and conduct full-scale trials to overcome issues related to imprecision and improve the robustness of the findings. Beyond stronger trial designs and adequate sample sizes, methodological rigour should also encompass approaches that better reflect the real-world circumstances of caregivers. This includes integrating Public and Patient Involvement to ensure that interventions are feasible, acceptable, and relevant to those who will ultimately use them. Among the included studies, only two indicated the inclusion of Public and Patient Involvement (Salehinejad et al., 2022; Windle et al., 2025). The limited inclusion of Public and Patient Involvement, or other involvement strategies, raises questions about whether the absence of it in studies may have influenced intervention outcomes. While it is not possible to judge to what extent this is the case, the inclusion of Public and Patient Involvement has been noted to be particularly important in dementia research, as it has been found to positively impacts research outcomes (Miah et al., 2019). Although the intervention developed by Windle et al. (2025) did not show superiority over usual care, their meaningful Public and Patient Involvement contributions, such as improving accessibility and implementation support, illustrate how involvement can enhance intervention relevance and feasibility. Finally, most of the studies included in this review were conducted in western counties. Although no language restrictions were applied during the database search, no eligible non-English publications were identified for inclusion. Among all the included studies, only one showed a statistically-significant effect on caregiver burden, and this was the only study conducted in a non-Western country (Salehinejad et al., 2022). Given the substantial impact of culture on caregiver experiences and coping processes (Pharr et al., 2014), the requirements, acceptance, and effectiveness of online psychosocial interventions among caregivers in non-Western countries may differ from those in Western countries.

### 4.3 Strengths and limitations

We conducted this review systematically and comprehensively in accordance with established guidelines. We have provided a focused synthesis of the current evidence by examining randomized controlled trials that compare online psychosocial interventions with usual care among informal caregivers of people with dementia. By concentrating on caregiver burden and self-efficacy, which are closely associated with caregiver well-being and the quality of care provided, we have offered a targeted and methodologically robust assessment of existing research in this area. We have also highlighted the design and content features of online psychosocial interventions, illustrated their diversity, and helped identify key gaps and limitations in current research, offering implications for future research and practice. By incorporating multiple perspectives, Patient and Public Involvement ensured a more nuanced and robust interpretation of the findings. However, our review had several limitations. First, by only including randomized controlled trials, we may have excluded potentially- informative studies, such as high-quality observational studies or quasi-experimental designs, which could provide additional insights into the effectiveness of online psychosocial interventions. Second, some studies were excluded from meta-analysis because of limited availability in data, despite efforts to obtain additional information by contacting the original investigators. Third, by focusing specifically on caregiver burden and self-efficacy, we may have excluded studies that examined conceptually- related constructs such as strain, stress, or competence. While this narrower focus allowed for greater conceptual clarity and comparability, it limited the scope of our synthesis. Nevertheless, burden and self-efficacy are key mechanisms that contribute to, but may not fully capture, caregivers’ overall well-being and quality of life. Researchers may benefit from developing intervention logics that reflect the existing theories, concept, and lived experience.

## 5 Conclusion

The central contribution of this article is a rigorous synthesis of online psychosocial interventions for informal caregivers of people with dementia, particularly focusing on burden and self-efficacy. Online psychosocial interventions feature diversity in their design, content, and delivery characteristics. While the overall certainty of the evidence is low, we have indicated that online psychosocial interventions have no short-term effect on burden but have a positive effect on self-efficacy. Further high-quality studies are essential to strengthen the evidence, particularly by addressing methodological limitations and ensuring rigorously designed interventions. Sustained support incorporating interactive and real-time elements, culturally tailored interventions, and patient and public involvement throughout the research process should be considered to improve both the effectiveness and relevance of these interventions.

## Funding

This work was supported by Alzheimer Society of Ireland [Person Public Involvement (PPI) Bursary, Postgraduate Research Bursary Scheme]; The first author is the recipient of a PhD Scholarship from China Scholarship Council [grant number 202106370226].

## Ethical approval statement

Ethical approval was not required for this systematic review, as it did not involve direct interaction with human participants or the collection of new primary data. Additionally, ethical approval was not required for Public and Patient involvement (PPI) in the interpretation of findings and dissemination stages of research.

## CRediT authorship contribution statement

**Qiuxia Li:** Writing – original draft, Methodology, Investigation, Funding acquisition, Formal analysis, Conceptualization. **Dou Zhang:** Writing – review & editing, Data curation. **Éle Quinn:** Writing – review & editing, Data curation. **Prof. Kieran Walsh:** Writing – review & editing, Supervision. **Dr. Sinéad Hynes:** Writing – review & editing, Validation, Supervision, Funding acquisition, Conceptualization.

## Declaration of competing interest

The authors declare that they have no known competing financial interests or personal relationships that could have appeared to influence the work reported in this paper.

## Appendix Supplementary materials

Image, application 1

Image, application 2
