# Supplementary material for: Online interventions for dementia caregiver burden and self-efficacy: A systematic review and meta-analysis
Source: Int J Nurs Stud Adv. 2026 Jan 22;10:100490. doi: 10.1016/j.ijnsa.2026.100490 (PMC12886064; doi:10.1016/j.ijnsa.2026.100490)
Supplement: Supplementary file 1 [file mmc1.docx]

**Supplementary files list**

Supplementary file 1: PRISMA flow diagram (Page et al., 2021)

Supplementary file 2: Risk of bias summary for burden: (A) individual studies; (B) overall

Supplementary file 3: Risk of bias summary for self-efficacy: (A) individual studies; (B) overall

Supplementary file 4: Forest plots: Effects of online psychosocial intervention on burden

Supplementary file 5: Forest plots: Effects of online psychosocial intervention on self-efficacy


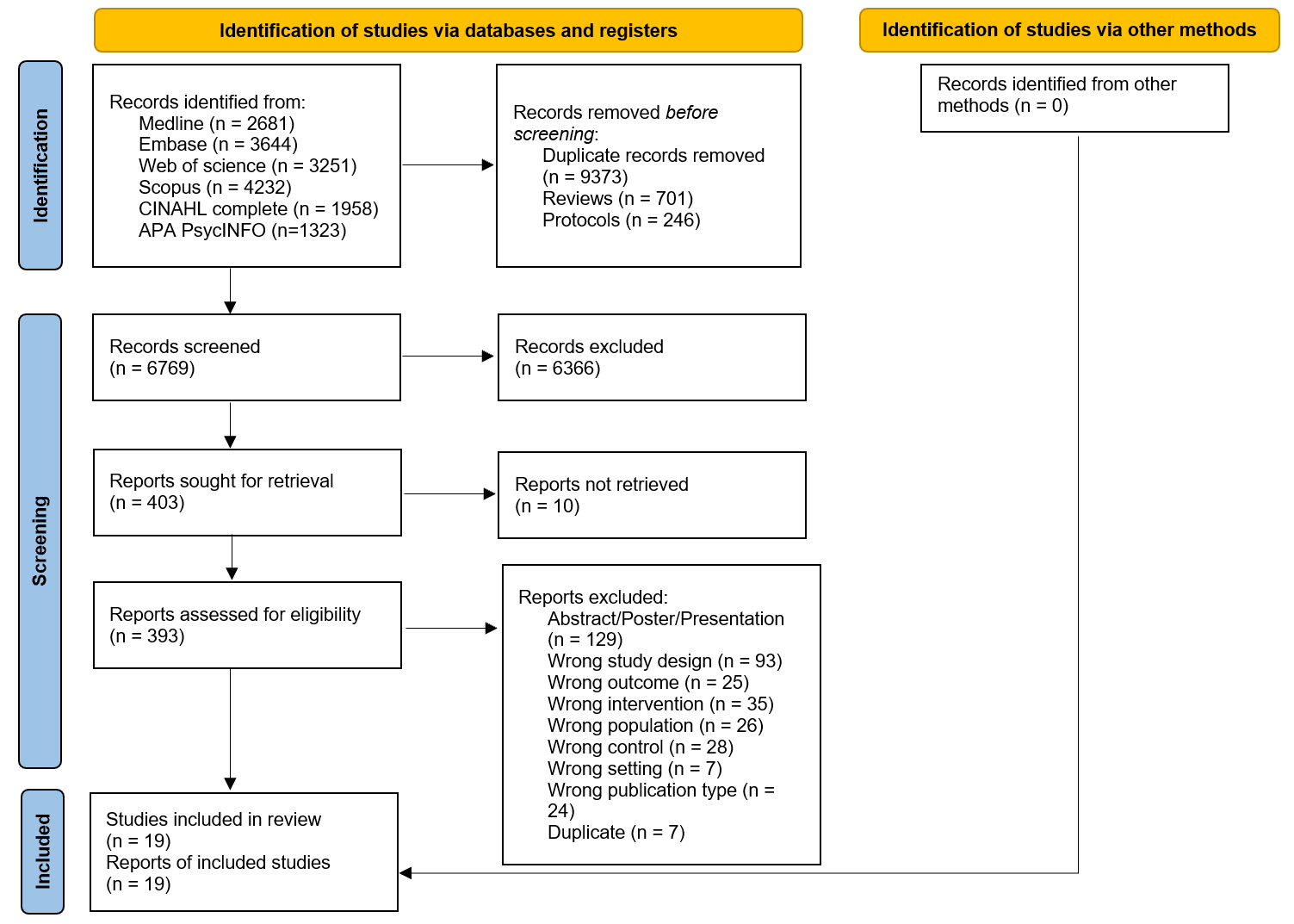


**Supplementary file 1: PRISMA flow diagram (Page et al., 2021)**

A


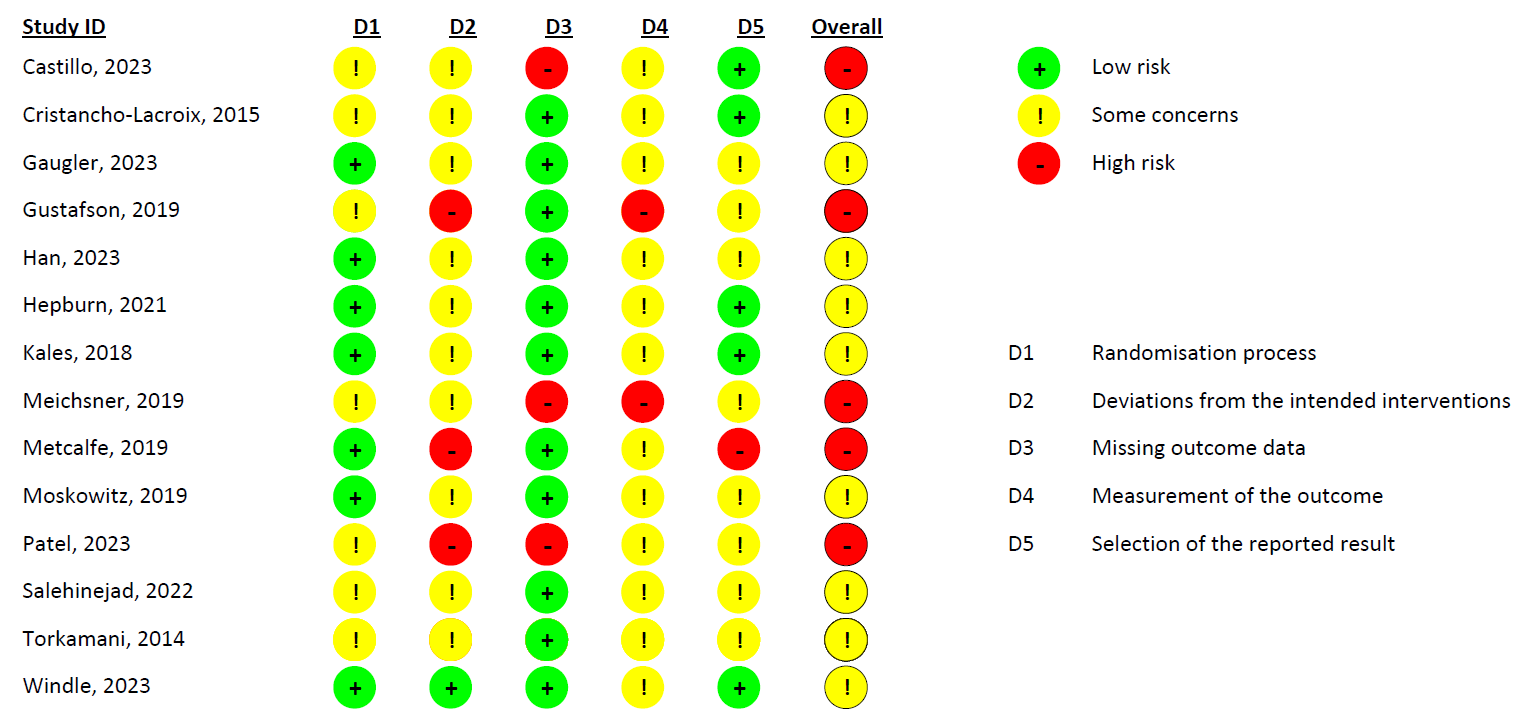


B


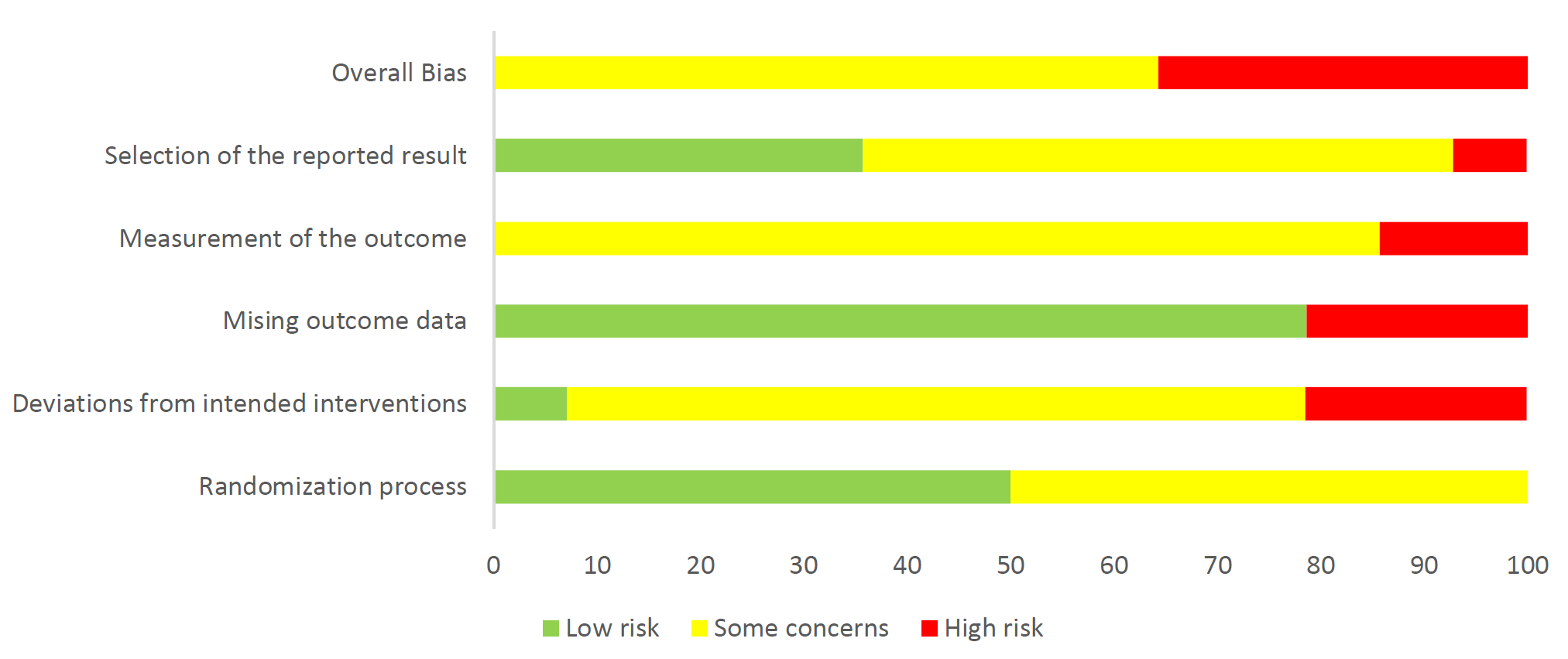


**Supplementary file 2: Risk of bias summary for burden: (A) individual studies; (B) overall.** *Blackberry et al., 2023*, a cluster-randomised controlled trial, was assessed separately using the RoB 2 tool for CRCTs and not included in the figure. Risk of bias ratings were: D1a (Randomisation process) – Low; D1b (Timing of identification/recruitment) – High; D2 (Deviations from intended interventions) – Low; D3 (Missing outcome data) – High; D4 (Measurement of outcome) – Some concerns; D5 (Selection of reported result) – Some concerns; Overall – High.

A


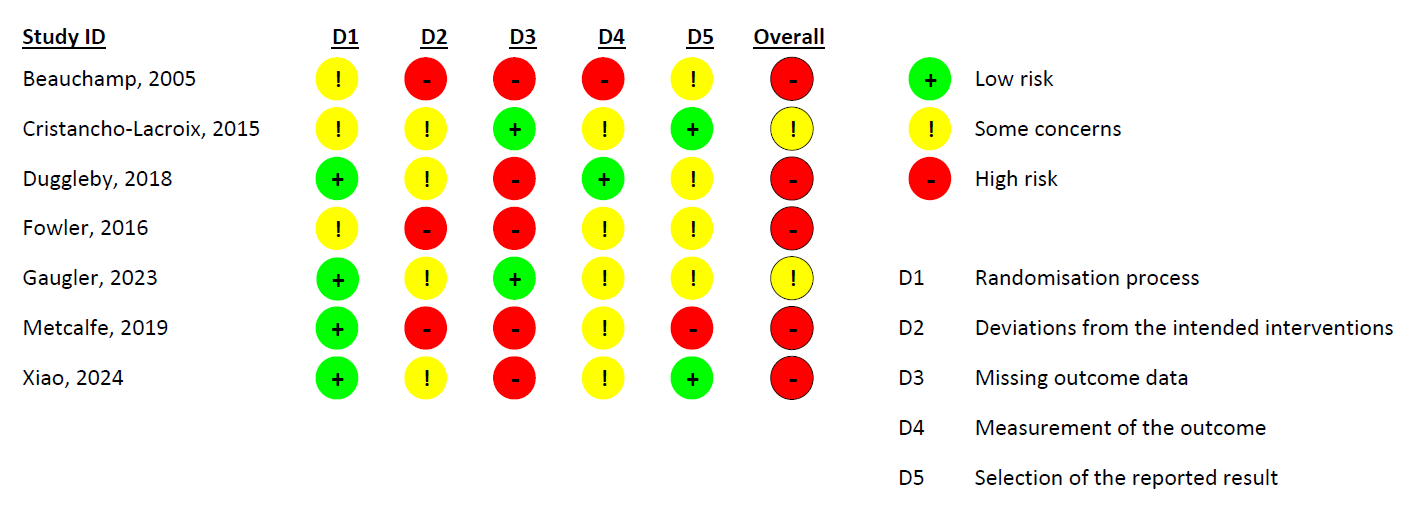


B


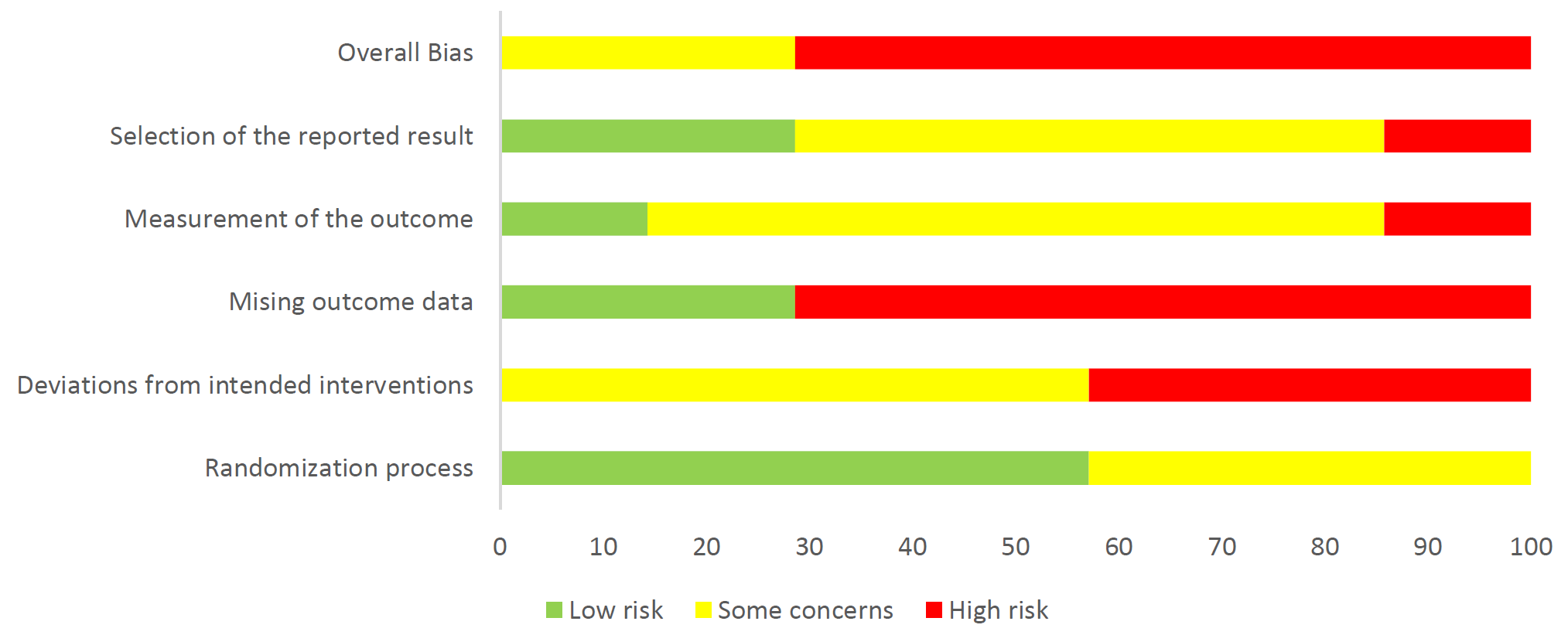


**Supplementary file 3: Risk of bias summary for self-efficacy: (A) individual studies; (B) overall**


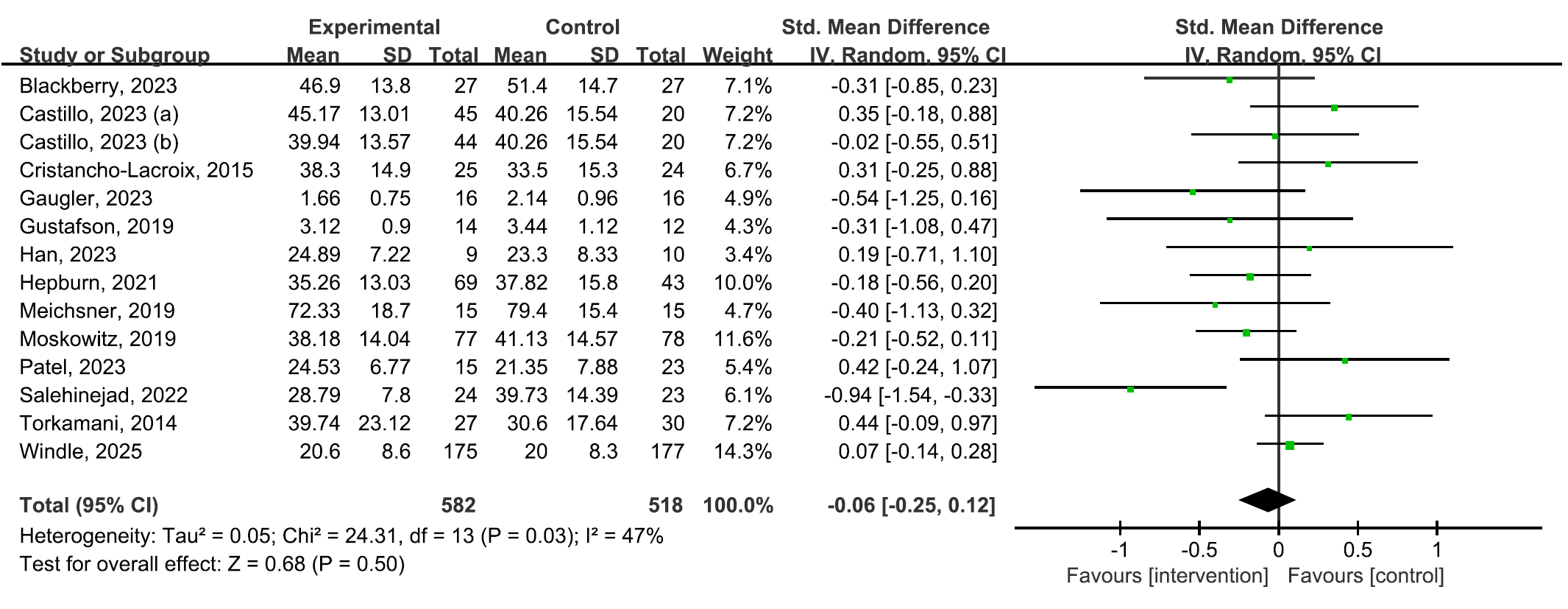


**Supplementary file 4: Forest plots: Effects of online psychosocial intervention on burden**


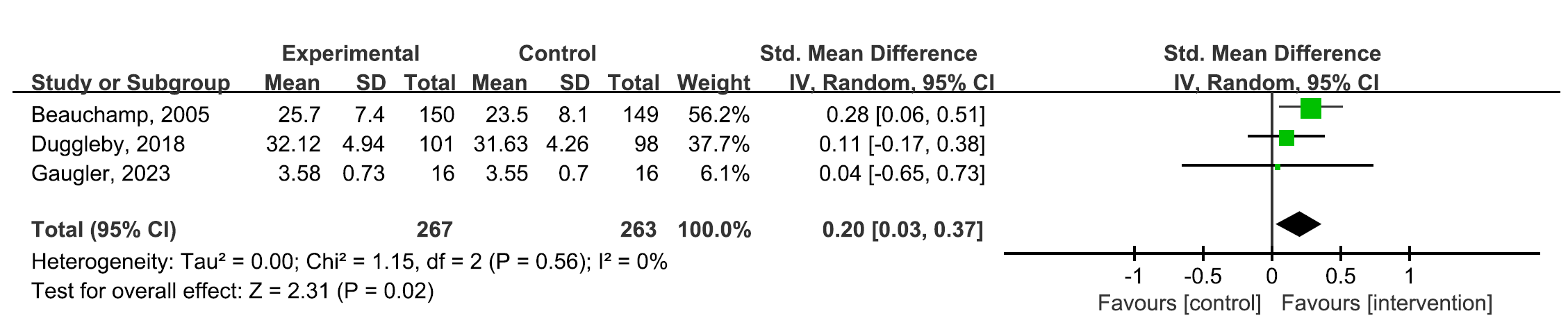


**Supplementary file 5: Forest plots: Effects of online psychosocial intervention on self-efficacy**
