# Supplementary material for: Online interventions for dementia caregiver burden and self-efficacy: A systematic review and meta-analysis
Source: Int J Nurs Stud Adv. 2026 Jan 22;10:100490. doi: 10.1016/j.ijnsa.2026.100490 (PMC12886064; doi:10.1016/j.ijnsa.2026.100490)
Supplement: Supplementary file 2 [file mmc2.docx]

**Supplementary files list**

Supplementary File 6: Search strategy

Supplementary File 7: Characteristics of care recipients of the included studies

Supplementary File 8: Health equity information of the participants based on the PROGRESS-Plus framework

Supplementary File 9: Detail intervention content of the included studies

Supplementary File 10: GRADE assessment

Supplementary File 11: PRISMA Checklist

**Supplementary File 6: Search strategy**

1. **Medline**

exp Caregivers/ or (caregiver* or carer* or care giver* or guardian* or caretaker*).mp.

AND

exp Dementia/ or (dementia or Alzheimer*).mp.

AND

exp Internet-Based Intervention/ or (Internet-Based or web-based or technology-based or online or on-line or ehealth or e-health or mHealth or m-Health or electronic* or mobile* or phone* or computer* or digital or telehealth or virtual or remote).mp.

1. **Embase**

'caregiver'/exp OR caregiver*:ti,ab,kw OR carer*:ti,ab,kw OR guardian*:ti,ab,kw OR 'care giver*':ti,ab,kw OR caretaker*:ti,ab,kw

AND

'dementia'/exp OR dementia:ti,ab,kw OR alzheimer*:ti,ab,kw

AND

'Internet-Based Intervention'/exp OR 'internet-based':ti,ab,kw OR 'web-based':ti,ab,kw OR 'technology-based':ti,ab,kw OR 'on-line':ti,ab,kw OR online:ti,ab,kw OR ehealth:ti,ab,kw OR 'e-health':ti,ab,kw OR 'm-Health':ti,ab,kw OR mHealth:ti,ab,kw OR electronic*:ti,ab,kw OR mobile*:ti,ab,kw OR phone*:ti,ab,kw OR computer*:ti,ab,kw OR digital:ti,ab,kw OR telehealth:ti,ab,kw OR virtual:ti,ab,kw OR remote:ti,ab,kw

1. **Web of Science**

TS=(caregiver* or carer* or “care giver*” or guardian* or caretaker*)

AND

TS=(dementia or Alzheimer*)

AND

TS=("Internet-Based" or "web-based" or "technology-based" or online or "on-line" or ehealth or “e-health” or mHealth or “m-Health” or electronic* or mobile* or phone* or computer* or digital or telehealth or virtual or remote)

1. **Scopus**

TITLE-ABS-KEY ( caregiver* OR carer* OR "care giver*" OR guardian* OR caretaker* )

AND

TITLE-ABS-KEY ( dementia OR alzheimer* )

AND

TITLE-ABS-KEY ( "internet-based" OR "web-based" OR "technology-based" OR online OR "on-line" OR ehealth or “e-health” or “m-Health” or mHealth OR electronic* OR mobile* OR phone* OR computer* OR digital OR telehealth OR virtual OR remote )

1. **CINAHL Complete**

| **#** | **Query** |
| --- | --- |
| S10 | S7 AND S8 AND S9 |
| S9 | S5 OR S6 |
| S8 | S3 OR S4 |
| S7 | S1 OR S2 |
| S6 | TI ( "Internet-Based" or "web-based" or "technology-based" or online or "on-line" or ehealth or "e-health" or mHealth or "m-Health" or electronic* or mobile* or phone* or computer* or digital or telehealth or virtual or remote ) OR AB ( "Internet-Based" or "web-based" or "technology-based" or online or "on-line" or ehealth or "e-health" or mHealth or "m-Health" or electronic* or mobile* or phone* or computer* or digital or telehealth or virtual or remote ) OR MW ( "Internet-Based" or "web-based" or "technology-based" or online or "on-line" or ehealth or "e-health" or mHealth or "m-Health" or electronic* or mobile* or phone* or computer* or digital or telehealth or virtual or remote ) |
| S5 | (MM "Internet-Based Intervention") |
| S4 | TI ( dementia or Alzheimer* ) OR AB ( dementia or Alzheimer* ) OR MW ( dementia or Alzheimer* ) |
| S3 | (MH "Dementia+") |
| S2 | TI ( caregiver* or carer* or "care giver*" or guardian* or caretaker* ) OR AB ( caregiver* or carer* or "care giver*" or guardian* or caretaker* ) OR MW ( caregiver* or carer* or "care giver*" or guardian* or caretaker* ) |
| S1 | (MM "Caregivers") |

1. **APA Psycinfo (EBSCOhost)**

| **#** | **Query** |
| --- | --- |
| S10 | S7 AND S8 AND S9 |
| S9 | S5 OR S6 |
| S8 | S3 OR S4 |
| S7 | S1 OR S2 |
| S6 | TI ( "Internet-Based" or "web-based" or "technology-based" or online or "on-line" or ehealth or "e-health" or mHealth or "m-Health" or electronic* or mobile* or phone* or computer* or digital or telehealth or virtual or remote ) OR AB ( "Internet-Based" or "web-based" or "technology-based" or online or "on-line" or ehealth or "e-health" or mHealth or "m-Health" or electronic* or mobile* or phone* or computer* or digital or telehealth or virtual or remote ) OR KW ( "Internet-Based" or "web-based" or "technology-based" or online or "on-line" or ehealth or "e-health" or mHealth or "m-Health" or electronic* or mobile* or phone* or computer* or digital or telehealth or virtual or remote ) |
| S5 | DE "Digital Interventions" |
| S4 | TI ( dementia or Alzheimer* ) OR AB ( dementia or Alzheimer* ) OR KW ( dementia or Alzheimer* ) |
| S3 | DE "Dementia" OR DE "AIDS Dementia Complex" OR DE "Alzheimer's Disease" OR DE "Dementia with Lewy Bodies" OR DE "Frontotemporal Lobar Degeneration" OR DE "Presenile Dementia" OR DE "Pseudodementia" OR DE "Senile Dementia" OR DE "Vascular Dementia" |
| S2 | TI ( caregiver* or carer* or "care giver*" or guardian* or caretaker* ) OR AB ( caregiver* or carer* or "care giver*" or guardian* or caretaker* ) OR KW ( caregiver* or carer* or "care giver*" or guardian* or caretaker* ) |
| S1 | (DE "Caregivers") |

**Supplementary File 7: Characteristics of care recipients of the included studies**

| **Author** | **Mean age (SD)** | **Gender** | **Dementia type** | **Dementia duration** |
| --- | --- | --- | --- | --- |
| **Beauchamp et al., 2005** | Not reported | Not reported | Not reported | Not reported |
| **Blackberry et al., 2023** | Not reported | Not reported | **Care recipient’s diagnosis, n (%)**  Dementia: 27 (72)  Cognitive impairment: 4 (10)  No formal diagnosis: 7 (18) | **Care recipient’s years since diagnosis, n (%)**  ＜2 years: 13 (34)  2-4 years: 5 (14)  ＞4 years: 9 (25) |
| **Castillo et al., 2023** | Not reported | Not reported | Not reported | Calculated in years [M (SD)]: IG1: 4.05 (2.86) IG2: 3.14 (4.30) CG: 3.53 (2.48) |
| **Cristancho-Lacroix et al., 2015** | Not reported | Not reported | Not reported | **Onset of symptoms (years), mean (SD), range** IG: 4.62 (3.53), 0.55-14.05 CG: 4.11 (3), 0.39-12.03 |
| **Duggleby et al., 2018** | IG: 80.5 (7.4) CG: 80.2 (8.0) | **Female, n (%)** IG: 46 (46) CG: 49 (50) | Not reported | Not reported |
| **Fowler et al., 2016** | IG: 85 (9.71) CG: 78 (8.64) | Not reported | Not reported | Not reported |
| **Gaugler, et al., 2023** | 79.51 (8.11) | **Female, n (%)** IG: 15 (34.9) CG: 15 (34.9) | Not reported | Not reported |
| **Gustafson et al., 2019** | Not reported | Not reported | Not reported | Not reported |
| **Han et al., 2023** | Not reported | Not reported | **Type of dementia in the relative with dementia, n (%)**  IG: Alzheimer’s disease: 6 (66.7), Not sure: 1 (11.1), Dementia with Lewy bodies: 1 (11.1), Vascular dementia: 1 (11.1)  CG: Alzheimer’s disease: 4 (40), Not sure: 3 (30), Dementia with Lewy bodies: 2 (20), Vascular dementia: 1 (10) | **Years since the relative’s diagnosis of dementia (years), mean (SD), range** IG: 3.6 (2.7), 1–10  CG: 4.4 (2.5), 1–9 |
| **Hepburn et al., 2021** | Active intervention group: 75.1 (8.6) Attention control group: 74.4 (10.6) Waitlist control group: 74.4 (10.3) | Not reported | Not reported | Not reported |
| **Kales et al., 2018** | IG: 82.3 (9.3) CG: 78.6 (10.8) | **Male, n (%)** IG: 8 (29) CG: 13 (43) | Not reported | Not reported |
| **Meichsner, et al., 2019** | IG: 72.47 (7.37)  CG: 77.5 (7.81) | **Female, n (%)**  IG: 8 (42.1)  CG: 9 (50.00) | **Type of dementia, n (%)**  IG: Alzheimer’s disease: 9 (47.4), Vascular dementia: 1 (5.3), Frontotemporal dementia: 3 (15.7), Other/unknown: 6 (31.6)  CG: Alzheimer’s disease: 11 (61.1), Vascular dementia: 1 (5.6), Frontotemporal dementia: 1 (5.6), Other/unknown: 5 (27.7) | Not reported |
| **Metcalfe et al., 2019** | IG: 61.6 (3.9) CG: 61.9 (5.7) | **Female, %** IG: 43.3 CG: 54.8 | **Alzheimer's disease/Frontotemporal degeneration, %** IG: 56.7/43.3 CG: 64.5/35.5 | **Years since diagnosis (1-2/3-4/5+), %** IG: 26.7/43.3/30 CG: 22.6/ 41.9/35.5 |
| **Moskowitz et al., 2019** | Not reported | Not reported | **Diagnosis, %** IG: Alzheimer's: 25.6, Frontotemporal Dementia: 12.8, Lewy Body Disease: 12.8, Parkinson's: 15.1, Other: 33.7 CG: Alzheimer's: 34.5, Frontotemporal Dementia: 9.5, Lewy Body Disease: 3.6, Parkinson's: 15.5, Other: 36.9 | Not reported |
| **Patel, et al., 2023** | Not reported | Not reported | Not reported | Not reported |
| **Salehinejad et al., 2022** | Not reported | Not reported | Not reported | Not reported |
| **Torkamani et al., 2014** | IG: 78.60 (7.49) CG: 77.47 (6.36) | **Male, %** IG: 45 CG: 55 | Not reported | **Years, Mean (SD)** IG: 3.7 (2.48) CG: 2.7 (1.73) |
| **Windle et al., 2025** | Not reported | Not reported | **Type of dementia (person cared for), n (%)**  IG: Alzheimer’s disease: 80 (45.7), Vascular Dementia: 28 (16.0), Familial Alzheimer’s Disease: 1 (0.6), Fronto-temporal Dementia: 4 (2.3), Primary Progressive Aphasia: 0 (0), Posterior Cortical Atrophy: 1 (0.6), Dementia with Lewy Bodies: 5 (2.9), Other: 46 (26.3), Don’t know: 10 (5.7)  CG: Alzheimer’s disease: 80 (45.2), Vascular Dementia: 26 (14.7), Familial Alzheimer’s Disease: 0 (0), Fronto-temporal Dementia: 5 (2.8), Primary Progressive Aphasia: 1 (0.6), Posterior Cortical Atrophy: 1 (0.6), Dementia with Lewy Bodies: 4 (2.3), Other: 47 (26.6), Don’t know: 13 (7.3) | Not reported |
| **Xiao et al., 2024** | IG: 79.5 (9.9)  CG: 77.4 (10.2) | **Female, n (%)** IG: 89 (67.9) CG: 80 (59.3) | **Type of dementia, n (%)**  IG: Alzheimer's disease: 63 (48.1), other types: 68 (51.9)  IG: Alzheimer's disease: 65 (48.1), other types: 70 (51.9) | Not reported |

Note: IG: Intervention group; CG: Control group.

**Supplementary File 8: Health equity information of the participants based on the PROGRESS-Plus framework** (O'Neill et al., 2014)

| **Author** | **PROGRESS-Plus: place of residence, race/ethnicity/culture/language, occupation, gender/sex, religion, education, socioeconomic status, social capital, personal characteristics associated with discrimination, features of relationships, time-dependent relationships** |
| --- | --- |
| **Beauchamp et al., 2005** | **Race, %** Caucasian: 80, African American: 4, Hispanic: 8, Other: 8 **Education, %** Some college or trade school: 90 |
| **Blackberry et al., 2023** | **Highest level of education, n (%)**  Secondary school (Year 7–Year 11): 13 (34)  Secondary school/TAFE/College: 15 (41)  Undergraduate tertiary education: 4 (10)  Postgraduate tertiary education: 5 (14) |
| **Castillo et al., 2023** | NA |
| **Cristancho-Lacroix et al., 2015** | **Education, n (%)** IG: High level: 19 (76), Middle level: 6 (24) CG: High level: 18 (75), Middle level: 3 (12) |
| **Duggleby et al., 2018** | **Marital Status, n (%)** IG: Married or living with someone: 84 (83), Single, widower, divorce or separated: 17 (17) CG: Married or living with someone: 85 (87), Single, widower, divorce or separated: 13 (13) **Race, n (%)** IG: Caucasian: 93 (92), Other: 8 (8) CG: Caucasian: 92 (94), Other: 6 (6) **Employment, n (%)** IG: Employed: 39 (39), Unemployed: 61 (61) CG: Employed: 47 (47), Unemployed: 50 (52) **Household incomes, n (%)** IG: Less than Can $40,000: 25 (29), Can $40,000 to $70,000: 23 (27),Greater than Can $70,000: 38 (44) CG: IG: Less than Can $40,000: 24 (30.4), Can $40,000 to $70,000: 16 (20.3),Greater than Can $70,000: 39 (49.3) |
| **Fowler et al., 2016** | **Area, n (%)** IG: Suburban: 11 (73), Urban: 4 (27) CG: Suburban: 9 (69), Uran: 4 (31) **Race, n (%)** IG: White: 13 (87), Black: NA, Asian/Hispanic/Hawaiian: 2 (13)  CG: White: 8 (62), Black: 3 (23), Asian/Hispanic/Hawaiian: 2 (15) **Income, n (%)** IG: <$20,000: 2 (20), $20,000 to $59,999: 4 (27),$60,000 to $99.999: 5(33), ≥$100,000: 2 (13),Prefer not to answer: 1(7) CG: <$20,000: 2 (15), $20,000 to $59,999: 5 (38),$60,000 to $99.999: 2 (15), ≥$100,000: 3 (23),Prefer not to answer: 1 (8) **Education, n (%)** IG: High school: 4 (27), Some college or 4-year degree: 8 (53), Graduate degree: 3 (20) CG: High school: 2 (15), Some college or 4-year degree: 5 (38), Graduate degree: 6 (46) **Employment, n (%)** IG: Not working: 1 (7), Working full- or part-time: 9 (60), Retired: 5 (39) CG: Not working: 8 (62), Working full- or part-time: 2 (15), Retired: 3 (23) |
| **Gaugler, et al., 2023** | **Race, n (%)**  White: 35 (81.4); Black/African American: 7 (16.3); Asian: 1 (2.3)  **Ethnicity, n (%)**  Hispanic or Latino: 0 (0)  **Education, n (%)**  High school diploma: 5 (11.6); Some college courses: 8 (18.6); Associate degree: 5 (11.6); Bachelor’s degree or higher: 25 (58.2)  **Income, n (%) (n=2 missing)**  $10,000 - $14,999: 1 (2.3); $10,000 - $14,999: 1 (2.3); $40,000 - $59,999: 4 (9.3); $60,000 - $79,999: 7 (16.3); ≥$80,000: 24 (55.8)  **Primary Caregiver, n (%)**  31 (72.1)  **Marital Status, n (%)**  Married and/or living with partner: 34 (79.1); Widowed: 3 (7.0); Divorced: 5 (11.6); Never married: 1 (2.3)  **Employment, n (%)**  Working full-time or part-time: 20 (46.5); Keeping house full-time: 1 (2.3); Retired: 18 (41.9); Unemployed: 1 (2.3); Other: 3 (7.0) |
| **Gustafson et al., 2019** | **Education, n (%)** IG: High school graduate: 2 (12.5), Some college: 3 (18.8), College graduate: 8 (50), Graduate degree: 3 (18.8) CG: High school graduate: 3 (20), Some college: 2 (13.3), College graduate: 7 (46.7), Graduate degree: 3 (20) |
| **Han et al., 2023** | **Race/Ethnicity, n (%)**  Black or African American: 9 (47.4)  Non-Hispanic White: 8 (42.1)  Hispanic or Latino: 1 (5.3)  Native American or American Indian: 1 (5.3)  **Level of education, n (%)**  High school graduate: 2 (10.5)  Technical, trade, or vocational school: 1 (5.3)  Some college: 5 (26.3)  Bachelor’s degree completed: 4 (21.1)  Postgraduate: 7 (36.8)  **Marital status, n (%)**  Married/Living with a partner: 8 (42.1)  Single/Never married: 6 (31.6)  Divorced/Separated: 4 (21.1)  Widowed: 1 (5.3)  **Employment status, n (%)**  Retired: 8 (42.1)  Employed full time: 6 (31.6)  Unemployed: 4 (21.1)  Employed part time: 1 (5.3)  **Religion, n (%)**  Christian: 14 (73.7)  Catholic: 2 (10.5)  No religion: 2 (10.5)  Jewish: 1 (5.3) |
| **Hepburn et al., 2021** | **Employment, n (%employed)** Active intervention group: 34 (35.8) Attention control group: 45 (40.9) Waitlist control group: 21 (38.9) **Education, n (% college graduate or higher)** Active intervention group: 41 (42.7) Attention control group: 46 (41.4) Waitlist control group: 21 (38.9) **Residence, n (%)** Active intervention group: Urban: 22 (22.9), Suburban: 55 (57.3), Rural: 19 (19.8) Attention control group: Urban: 49 (44.5), Suburban: 40 (36.4), Rural:21 (19.1) Waitlist control group: Urban: 22 (41.5), Suburban: 21 (39.6), Rural: 10 (18.9) **Race, n (%)** Active intervention group: Black: 15 (15.6), Asian: 1 (1), White: 78 (81.3), Did not answer: 0 (0), Other: 2 (2.1) Attention control group: Black: 27 (24.3), Asian: 3 (2.7), White: 75 (67.6), Did not answer: 1 (0.9), Other: 5 (4.5) Waitlist control group: Black: 15 (27.8), Asian: 0 (0), White: 39 (72.2), Did not answer: 0 (0), Other: 0 (0) **Ethnicity, n (%)** Active intervention group: Hispanic: 3 (3.1), Non-Hispanic: 92 (95.8), Did not answer: 0 (0) Attention control group: Hispanic: 7 (6.3), Non-Hispanic: 104 (93.7), Did not answer: 0 (0) Waitlist control group: Hispanic: 0 (0), Non-Hispanic: 54 (100), Did not answer: 0 (0) |
| **Kales et al., 2018** | **Marital Status, n (%)** IG: Single: 13 (48), Married: 14 (52) CG: Single: 11 (37), Married: 19 (63) **Race, n (%)** IG: White: 18 (67), African American: 7 (25), Other: 2 (7) CG: White: 19 (63), African American: 10 (33), Other: 1 (3) |
| **Meichsner et al., 2019** | **Education, n (%)**  IG: Primary or other: -, Secondary (level 2): 5 (26.3), Secondary (levels 3 & 4): 3 (15.8), Tertiary (levels 5 & 6): 11 (57.9)  CG: Primary or other: -, Secondary (level 2): 6 (33.3), Secondary (levels 3 & 4): 6 (33.3), Tertiary (levels 5 & 6): 6 (33.3) |
| **Metcalfe et al., 2019** | **Higher education, %** IG: 46.7 CG: 41.9 **Full/part time employment, %** IG: 50.0 CG: 67.7 **Retired, %** IG: 23.3 CG: 22.6 |
| **Moskowitz et al., 2019** | **Race, %** IG: Black/African American: 0, White/European: 91.9, Asian/Asian-American/Pacific Islander: 5.8, American Indian/Eskimo: 1.2, Mixed/Other: 1.2 CG: Black/African American: 4.8, White/European: 84.5, Asian/Asian-American/Pacific Islander: 8.3, American Indian/Eskimo: 0, Mixed/Other: 2.4 **Rurality, %** IG: Urban: 48.8, Suburban: 32.6, Rural: 18.6 CG: Urban: 52.4, Suburban: 32.1, Rural: 15.5 **Education, %** IG: <High School: 1.2, High School: 8.1,Some College: 18.6, College Graduate: 20.9, Associate Degree: 5.8, Some graduate school: 11.6, Masters: 19.8, Some post Masters work: 5.8, PhD, MD, JD Other: 8.1 CG: <High School: 0, High School: 3.6,Some College: 10.7, College Graduate: 29.8, Associate Degree: 13.1, Some graduate school: 8.3, Masters: 20.2, Some post Masters work: 1.2, PhD, MD, JD Other: 13.1 |
| **Patel, et al., 2023** | **Ethnicity, n (%)**  IG: White British (including Welsh/Scottish/English): 15 (100)  CG: White British (including Welsh/Scottish/English): 24 (100)  **Marital Status, n (%)**  IG: Married: 10 (66.67), Single: 3 (20), Living with Partner: 1 (6.67), Separated: 1 (6.67), Divorced: 0 (0)  CG: Married: 16 (66.67), Single: 1 (4.17), Living with Partner: 6 (25), Separated: 0 (0), Divorced: 1 (4.17) |
| **Salehinejad et al., 2022** | **Education, n (%)** IG: Primary education: 2 (8), Diploma and associate degree: 9 (36), Graduate: 8 (32), Postgraduate: 6 (24) CG: Primary education: 5 (20), Diploma and associate degree: 8 (32), Graduate: 8 (32), Postgraduate: 4 (16) **Employment, n (%)** IG: Employed: 11 (44), Unemployed: 14 (56) CG: Employed: 9 (36), Unemployed: 16 (64) **Income, n (%)** IG: Yes: 20 (80), No: 5 (20) CG: Yes: 19 (76), No: 6 (24) **Marital Status, n (%)** IG: Married: 21 (84), Single: 4 (16) CG: Married: 21 (84), Single: 4 (16) |
| **Torkamani et al., 2014** | NA |
| **Windle et al., 2025** | **Main language, n (%)**  IG: English: 172 (98.3), Welsh: 2 (1.1), Gaelic: 0 (0), Other: 1 (0.6)  CG: English: 175 (98.9), Welsh: 0 (0), Gaelic: 0 (0), Other: 2 (1.1)  **Ethnicity, n (%)**  IG: White English/Welsh/Scottish/Northern Irish/British: 164 (93.7), Irish: 1 (0.6), Any other White background: 3 (1.7), Indian: 1 (0.6), Chinese: 0 (0), Any other Asian background: 1 (0.6), White and Asian: 0 (0), Any other Mixed/multiple ethnic background: 0 (0), Caribbean: 3 (1.7), Any other Black/African/Caribbean background: 1 (0.6), Any other ethnic group: 1 (0.6), Prefer not to say: 0 (0)  CG: White English/Welsh/Scottish/Northern Irish/British: 164 (92.7), Irish: 0 (0), Any other White background: 4 (2.3), Indian: 2 (1.1), Chinese: 1 (0.6), Any other Asian background: 0 (0), White and Asian: 2 (1.1), Any other Mixed/multiple ethnic background: 2 (1.1), Caribbean: 1 (0.6), Any other Black/African/Caribbean background: 0 (0), Any other ethnic group: 0 (0), Prefer not to say: 1 (0.6)  **Level of education, n (%)**  IG: University Higher Degree (MA; MSc; PhD): 39 (22.3), First degree level qualification (BA; BSc): 62 (35.4), Apprenticeship: 4 (2.3), HND; HNC; NVQ Level 4; teaching; nursing: 21 (12.0), AS, A Level, Baccalaureate: 13 (7.4), NVQ level 3 or below, BTEC, City and Guilds Craft: 6 (3.4), Any other qualification: 11 (6.3), None of the above: 2 (1.1)  CG: University Higher Degree (MA; MSc; PhD): 47 (26.6), First degree level qualification (BA; BSc): 46 (26.0), Apprenticeship: 0 (0), HND; HNC; NVQ Level 4; teaching; nursing: 27 (15.3), AS, A Level, Baccalaureate: 14 (7.9), NVQ level 3 or below, BTEC, City and Guilds Craft: 2 (1.1), Any other qualification: 17 (9.6), None of the above: 3 (1.7) |
| **Xiao et al., 2024** | **Marital status: n (%)**  IG: Married: 75 (57.3), Unmarried/divorced/widowed: 56 (42.7)  CG: Married: 77 (57.0), Unmarried/divorced/widowed: 44 (43.0)  **Employment status: n (%)**  IG: Employed: 65 (49.6), Unemployed/retired: 66 (50.4)  CG: Employed: 69 (51.1), Unemployed/retired: 66 (48.9)  **Education level: n (%)**  IG: High school and below: 35 (26.7), Above high school: 96 (73.3)  CG: High school and below: 38 (28.1), Above high school: 97 (71.9) |

Note: Terminology used in this table is based on the terminology presented by the respective authors in the referenced papers; IG: Intervention group; CG: Control group; NA: Not applicable.

**Supplementary file 9: Detail intervention content of the included studies**

| **Authors** | **Content** |
| --- | --- |
| **Beauchamp et al., 2005** | 1. Individualized Tailoring. -The program offers personalized content through a questionnaire and tailored input based on the viewer's situation and their relationship to the care recipient's level of dementia.  2. Multiple Components. -The components of knowledge, cognitive, and behavioural skills, and affective learning. The coping strategies presented in all three modules emphasize problem-focused techniques and social support skills.  3. Being a Caregiver. -This module focuses on educating the caregiver about common caregiving issues.  4. Coping With Emotions. -This module focuses exclusively on affective issues, with video testimonials of caregivers discussing cognitive and behavioral strategies that have helped them deal with common emotions such as guilt, anger, grief, resentment, fear, anxiety, and helplessness.  5. Common Difficulties. -This module addresses common concerns and is tailored by the severity of dementia. |
| **Blackberry et al., 2023** | 1. An integrated website and mobile app (Verily Connect app). The Verily Connect app had 2 main functions: information provision and facilitation of social communication between users. General information relevant to a carer of a person living with dementia was provided by 12 *guides*. The guides were developed by the research team; they were deliberately brief and curated from freely accessible but reputable internet sources. Links to information sources were accessible on the app and could be clicked to open the linked source in a web browser. For each participating community, there was a directory of locally available dementia-relevant services that were geographically displayed using Google Maps. Service information included links within the app that directly connected app users to the telephone, email, Facebook, and website of the listed service (where available). In addition, the Verily Connect app provided opportunities for app users to connect with each other using a text-based chat function presented as *forums*. During the trial, to control when communities had access to the Verily Connect intervention, access to the Verily Connect app was password-protected; the research team gave participants access to a password and the app when their community entered the intervention phase.  2. Carer peer support groups that met via Zoom (Zoom Video Communications) videoconference. The project manager facilitated the implementation of the support groups by providing technical assistance and information about the group and videoconference etiquette (including precautions about privacy, confidentiality, and being secure when using the internet), making introductions, and ensuring that all members were given the opportunity to contribute to discussions. Most groups did not have a specific agenda; rather, the participants could speak about whatever they wanted. The project manager used minimal questioning and prompting to encourage conversation and ensure that every participant had a turn to speak. A challenge sometimes arose if a participant had poor internet connectivity and, therefore, had trouble keeping up with the group conversation. Another challenge arose when only one of the group members joined by telephone while the others were on videoconference; the person on the telephone missed nonverbal cues provided by those on videoconference and, consequently, there were some miscommunications and frustration with mistiming of discussions. The first carer peer support group was held at the end of wave 2 as this length of time was needed for sufficient carers to be recruited and their communities to enter the intervention phase. Thereafter, the carer peer support groups met monthly. Attendance at carer peer support groups was managed by direct invitation to participants whose communities had entered the intervention phase.  3. Volunteer support and a *Technology Learning Centre* (also known as a Verily Connect Hub) that was physically located in each community. The role of the volunteers was to assist carers and other interested community members in learning how to use the Verily Connect app and other relevant web-based technologies (such as Zoom videoconferencing). Volunteers were governed by a health service or volunteer organization in their local community, and they received a day’s training from Verily Connect project staff. The Verily Connect project also facilitated support for volunteers via group videoconference meetings. Verily Connect Hubs were slightly different in each community; however, each community was given an iPad (Apple Inc) and Samsung S4 phone and an Aus $2000 (US $1419.60) budget to purchase resources for the Hub, such as books about dementia, web cameras, headsets, tablets, and items to assist people living with dementia (eg, simplified clocks, therapy dolls, and activities for people living with dementia). The Hubs were established, and volunteers received their training only when the community entered the intervention phase. |
| **Castillo et al., 2023** | **IG1: (Dementia Talk):**  Features:  1. Medication management  2. Task and appointment tracking  3. Stress management activities  4. Information about managing care and caregiver well-being  5. Behaviour/symptom managing  6. Task management with other care providers  7. External resources  Components found in theoretical frameworks of stress: coping strategies, objective indicators of stress, subjective indicators of stress, and social support.  The app provided users with information about common behaviours (e.g., apathy, irritability, sleeping problems) that may be observed in the person living with dementia, as well as allowed users to track the frequency and intensity of the observed behaviour. Moreover, the app allowed users to choose strategies to manage a chosen behaviour. The Dementia Talk app included information related to stress and its management. For example, the app included information about caregiver stress, tips to manage it, and a video clip of a relaxation exercise for the caregiver. The app also included features that allows users to schedule and manage tasks and medication. Other individuals in the care team can be added to the app to facilitate provision of care.  **IG2 (CLEAR Dementia Care):**  Features:  1. Information on dementia  2. Behavior/symptom monitoring  Components found in theoretical frameworks of stress: coping strategies, objective indicators of stress.  The app provided information about dementia, the different types of dementia, how dementia affects the brain, and symptoms associated with dementia. In addition, the app provided information on five domains (e.g., cognition, life story and personality, relationships, activity and environment, emotional and physical wellbeing) to facilitate an understanding of the experiences of the person living with dementia. The app offered suggestions and alternative approaches to various care-related situations (e.g., repetitive questions, personal care) through illustrations/images that caregivers may experience. The app also included a feature that allows users to record the frequency of an observed behaviours. |
| **Cristancho-Lacroix et al., 2015** | **Weekly Sessions:**  **One session per week had to be entirely viewed at least once to unblock the next session**  Session 1. Caregiver stress  Session 2. Understanding the disease  Session 3. Maintaining the loved ones’ autonomy  Session 4. Understanding their reactions  Session 5. Coping with behavioural and emotional troubles  Session 6. Communicating with loved ones  Session 7. Improving their daily lives  Session 8. Avoiding falls  Session 9. Pharmacological and nonpharmacological interventions  Session 10. Social and financial support  Session 11. About the future  Session 12. In a nutshell  **Other sections:**   1. Relaxation training: guidelines for learning relaxation as well as 2 videos for the modelling of Schultz’s Autogenic Training and Jacobson’s method. 2. Life Stories: stories about 4 couples, based on testimonials of caregivers, in which difficult situations are illustrated and possible solutions to manage them are discussed (eg, apathy of patient, caregivers’ isolation). 3. Glossary: a glossary for technical words (eg, neuropsychological assessment, aphasia) 4. Stimulation: practical activities to stimulate autonomy and share pleasant activities with the relatives in daily life. 5. Forum: a private and anonymous forum to interact with peers, to express their concerns, discuss solutions to daily problems, and share their feelings and experiences. The participants use nicknames to protect their privacy. A clinical psychologist participates in the discussions if necessary (ie, avoiding aggressive or inappropriate comments). |
| **Duggleby et al., 2018** | **MT4C consists of six main sections:**  (1) about me: My story; What helps me; My goals as care partner; What is my back-up plan; Everyday hope; What am I doing for myself today;  (2) common changes to expect;  (3) frequently asked questions;  (4) resources: Your contact list; National contacts; Helpful Resources; Planning;  (5) important health information,  and (6) calendar. |
| **Fowler et al., 2016** | The VHN is an asynchronous website that provides social support through a blog, specific educational material, and the opportunity to ask questions of the interprofessional team participating in this project.  **Educational material including the following topics:**  1. Social support-What it is and why it is important  2. Social support- Types of support  3. Social support- How to improve my network  4. Social support and your health  5. Communication  6. Home safety  7. Tips for helping with activities of daily living  8. Behaviour concerns  9. Sleep and the caregiver  10. Sleep and understanding the actigraphy band  11. Sleep and the person with dementia  12. Movement and the person with dementia- Part 1  13. Movement and the person with dementia- Part 2  14. Community resources  15. Oral health and the caregiver  16. Gum disease and dentures  Blog, ‘Ask the Expert’ allowing caregivers to have a one-on-one discussion with investigators |
| **Gaugler, et al., 2023** | **CtP includes three elements:**  (a) a brief, 20-item assessment of need on the part of the caregiver and care recipient with dementia (Czaja et al., 2009);  (b) generation of personalized support recommendations for the dementia caregiver;  Recommendations are mapped to seven types of interventions:   1. Psychoeducation 2. Case management or counselling 3. Support groups 4. Respite services 5. Cognitive rehabilitation for the care recipient 6. Psychotherapy for caregivers 7. Multicomponent approaches   and (c) provision of local resources to facilitate the caregiver’s selection of a recommended support option. |
| **Gustafson et al., 2019** | **Home page:** 1. Thought of the Day: Prompt that changes daily, targeting common concerns of caregivers (e.g., anxiety, financial issues, healthy eating) with links to resources. (prompts, family support, quality information)  **Reading Room area:** 1. Library Topics: Extensive on-site collection of informational readings on topics related to dementia and caregiving created by experts at UW, and links to relevant external websites vetted for quality. (action planning, family support, quality information) 2. FAQs: Searchable list of frequently asked questions and answers about dementia and related diseases, self-care and stress reduction, symptom and behavior management, financial and legal issues, medical care, end of life, and more. (action planning, family support, quality information) 3. Personal Stories: Real-life accounts from caregivers of family members with dementia, with prompts to explore 1) coping techniques, 2) in-depth descriptions of patient behaviors, symptoms, and caregiving stress, and 3) advice based on experiences. (peer support, family support, prompts) 4. Caregiver Tips: Quick tips from professional sources covering a broad range of issues, such as coping with the emotional side of caregiving, relating to the patient, and helping with everyday activities. Study participants also share their own tips. (peer support, family support, prompts, quality information)  **Support area:** 1. Discussion Group: Private, interactive online discussion group for caregivers of dementia patients for sharing and soliciting information, ideas, experiences, and support. (assertive outreach, peer support, family support, prompts) 2. My Journal: Private journaling feature for caregivers, with prompts for writing and reflection. (prompts, action planning) 3. Easing Distress: Collection of cognitive-behavioral, psychological, relaxation, and lifestyle strategies for coping with distress, including audio meditations, cognitive reframing exercises, and more. (prompts, action planning, peer support, quality information)  **Tools area:** 1. Weekly Check-In: Weekly caregiver report on measures of their own health and well-being, with responses tracked over time. When appropriate (e.g., indications of depression), specialists at UW follow up. (assertive outreach, monitoring, prompts, case management) 2. Placement Decision Guide: Step-by-step decision guide to help caregivers clarify situations and needs when considering placing a family member in specialized care. (prompts, action planning, family support) 3. Respite Planner: Interactive tool for 1) thinking through potential benefits and barriers to respite and 2) respite planning. (prompts, action planning, family support) 4. Action Planner: Interactive tool for creating a step-by-step plan for any change for caregiver or patient, such as bringing in help or changing doctors, establishing a budget, starting a relaxation routine, and more. (prompts, action planning, family support)  **Finding Help area:** 1. Ask a Specialist: Private two-way messaging between caregivers and Alzheimer’s information specialists: 1) caregivers reach out at any time to request information and advice, 2) specialists initiate support when caregivers show need (e.g., depression) via responses on bi-monthly paper surveys. (assertive outreach, prompts, case management, family support, quality information) 2. Family & Friends: Updates and private messaging for family and friends of caregivers and their patients, available via sign-up. (assertive outreach, prompts, peer support, family support) 3. Community Resources: Alphabetical listing of health, social service, and other community organizations, as well as relevant websites, providing information and support to caregivers of dementia patients. (peer support, family support, quality information)  **External sensors:** 1. GPS Tracking: Location-monitoring via a tracker worn by the patient. (monitoring, family support) 2. Caregiver Alerts: Motion-activated sensors strategically placed (e.g., on doors) that notify caregivers when triggered. (monitoring, family support) 3. Item Finder: Bluetooth trackers, attached to items frequently lost, that emit a sound via a smartphone app when the item is sought. (monitoring, family support) |
| **Han et al., 2023** | **8 weekly acceptance and commitment therapy (ACT) sessions:**  Week 1. Orientation and identifying difficulties in ACT processes  - Orienting the caregiver to the intervention sessions  - Conducting an initial interview to identify the caregiver’s difficulties in six ACT processes  - Introducing basic concepts of ACT using metaphors  *Learning materials:* ACT initial case conceptualization form & ACT in a nutshell metaphor  Week 2. ACT (Creative Hopelessness)  - Helping the caregiver explore the efforts he or she has made to try to eliminate, change, or fix his or her negative internal experiences (i.e., difficult thoughts and feelings)  - Guiding the caregiver in assessing the workability of these efforts  *Learning materials:* My coping strategies worksheet and metaphors  *Homework assignment*: Creative hopelessness worksheet and guided online ACT exercises  Week 3. ACT (Control vs. Willingness)  - Helping the caregiver acknowledge and consider experiencing the uncomfortable and unwanted emotions and thoughts as they are as an alternative to control  - Guiding the caregiver in exploring barriers to willingness and practicing exercises to facilitate overcoming barriers  *Learning materials:* Metaphors, contacting my painful emotions worksheet  *Homework assignment:* Control as the problem worksheet, willingness worksheet, and guided online ACT exercises  Week 4. ACT (Acceptance)  - Fostering the caregiver’s willingness/acceptance using the case scenario and analysis exercise  - Coaching the caregiver in learning and applying strategies adapted to the caregiver’s patterns of experiential avoidance  *Learning materials:* Case scenario exercise and coping with grief worksheet  *Homework assignment:* Guided online ACT exercises  Week 5. ACT (Cognitive defusion, observing self, and being present)  - Coaching the caregiver in: learning and applying exercises to step back or detach from unhelpful thoughts and emotions; practicing exercises to observe thoughts and emotions without judgement; and learning and practicing mindfulness exercises to promote maintaining contact with the present moment and living in the “here and now”  *Learning materials:* Cognitive defusion exercises (e.g., passengers on the bus metaphor), exercises to observe thoughts and emotions (e.g., the continuous you/observer exercise), and mindfulness exercises (e.g., mindful breathing and body scan)  *Homework assignment:* Self-as-context worksheet and guided online ACT exercises  Week 6. ACT (Values and committed action) + Behavioral activation (BA)  - Assisting the caregiver with clarifying the core values that give them meaning/purpose and identifying potential barriers to living a values-focused life; identifying ways to overcome these barriers; setting a committed action plan; and using BA techniques (activity scheduling and monitoring) for involvement during week 6 in committed actions aligned with the caregiver’s personal values  *Learning materials:* Exercises to help clarify values (e.g., tombstone exercise), demons on the boat metaphor, reflecting on your values, and my action plan worksheets  *Homework assignment:* Values worksheet and activity scheduling and monitoring worksheet  Week 7. ACT (Values and committed action) + BA  - Reviewing the caregiver’s performance of committed actions and mood in the past week and discussing barriers that hindered their committed actions during this period and ways to overcome those barriers and assisting the caregiver in revising their committed action plan for values-based living  - Coaching the caregiver in the use of mindfulness and defusion skills to follow through on the committed actions in the face of negative/painful thoughts and feelings  *Learning materials:* My action plan worksheet  *Homework assignment:* Activity scheduling and monitoring worksheet & guided online ACT exercises  Week 8. ACT + BA and Closure  - Same as the week 7 plus wrap up  *Learning materials*: Joe the problem metaphor and my action plan worksheet  *Selected ACT online resources were provided after the completion of session 8 |
| **Hepburn et al., 2021** | Tele-Savvy emphasizes the development of knowledge, skills and outlook, and caregiving mastery as means to engage successfully in the ‘unexpected career’ that caregivers have undertaken. Tele-Savvy is delivered over 43 days to groups of 6–8 caregivers in 7 weekly synchronous sessions accompanied by 36 brief asynchronous video lessons. Each videoconference (after the first) begins with a review of the home assignments that were given.  **What is the Tele-Savvy Program About?**  1. Guiding Daily Life. This area makes up the majority of Tele-Savvy content. The first three weeks of the program deal, in sequence, with the impact of progressive dementia on the person living with the illness. The material in the videoconferences and video lessons takes caregivers through the ways in which these diseases affect thinking, feeling, behavior, and the ability to do things in life.  2. Guiding Behavior. The program attempts to give caregivers a basis for understanding behaviors they might consider difficult or troubling. Moreover, it provides strategies for quelling and/or responding to the behaviors so that they reduce in intensity or stop or so that the caregiver finds them less troubling.  3. Managing Personal Life and Well-Being. The program examines the impact of caregiving on caregivers and provides tools for caregivers to examine their own feelings (especially those that are negative and reinforce the sense that they are powerless in the situation) and to do something about them. The program also urges caregivers to examine their own interests and to have a repertoire of things that they will do when they can free up time to do them. Content on mindfulness practices has been added in Tele-Savvy. The section of the program focusing on decision-making is linked to this area of caregivers’ work.  4. Managing Resources. Content about this topic is in two parts. The first centers on the family as a resource for caregiving. The second area of content involves two video lessons centered on mapping the caregiver’s resource environment. |
| **Kales et al., 2018** | 1. The first components is a guided DICE approach where a peer navigator (tailored to the age, race and gender of the caregiver) leads the caregiver through the approach; this occurs through the caregiver answering questions related to symptom context (who, what, when, where) and possible medical/pain issues  including delirium. Based upon these answers, an algorithm selects from over 900 evidence-based strategies to create a WeCareAdvisor ‘prescription’. Caregivers are instructed to try the strategies for one week and then evaluate how the strategies work for them; if the strategies are helpful, they are encouraged to keep using them, if the strategies have not been helpful, caregivers are encouraged to conduct another DICE session to get a new set of strategies. During orientation, caregivers conducted their first DICE session on the most problematic behaviour they were experiencing, and during their one-month trial were encouraged to create as many sessions as they feel would be helpful to them.  2. The second component is Caregiver Survival Guide which is a compendium of information for dementia caregivers (e.g. ‘what is dementia’, ‘keeping the person with dementia healthy’);  3. The third component is a daily messaging feature that provides an encouraging daily communication to caregivers for support and motivation. |
| **Meichsner et al., 2019** | The manual follows cognitive‐behavioural principles and consists of 10 therapy modules:  1. Basic elements: Creating therapeutic alliance, structuring each message, handling crises  2. Problem analysis: Individual problem analysis to identify the participant’s main problem areas  3. Psychoeducation: Therapist provides information on caregiving, dementia, and general psychological processes and concepts (e.g., acceptance)  4. Strengthening problem‐solving abilities: Problem‐solving training to develop and support the participant’s individual problem‐solving process. Often applied with regard to coping with challenging behavior of the care recipient  5. Changing dysfunctional cognitions: Socratic questions and guided discovery are used to identify dysfunctional beliefs and to develop more helpful ways of thinking  6. Increasing the use of informal and/or professional support: Utilizing (more) professional and informal help is addressed as well as allowing and accepting help  7. Coping with change, grief, and loss: Emotion‐based coping‐strategies and acceptance of the disease and the resulting changes are addressed  8. Self‐care, creating value‐based activities: Activities to improve self‐care (e.g., value‐based positive activities, health‐promoting activities) are identified. Implementation of identified activities into daily life. Addressing negative feelings such as guilt while increasing value-based behavior  9. Stress‐management and emotion regulation strategies: Accepting emotions such as anger. Developing emotion regulation strategies. Decreasing inner tension  10. Evaluation: Achieved changes are summarized, plans for the participant’s future are made |
| **Metcalfe et al., 2019** | The RHAPSODY project (Research to Assess Policies and Strategies for Dementia in the Young):  **Seven modules:**  1. The nature of young‐onset dementia;  2. Medical explanations;  3. Common problems and solutions;  4. Management of cognitive and behavioral symptoms;  5. Adapting to relationship changes;  6. Available care and support;  7. Self‐care suggestions.  The multimedia format combines written and video content, case‐studies, presentations from professionals, and downloadable materials. |
| **Moskowitz et al., 2019** | The positive emotion regulation intervention (LEAF) consisted of 6 sessions in which a facilitator taught participants a set of 8 emotion regulation skills intended to increase positive emotion.  1. In session 1, the facilitators presented the first three skills: noticing positive events, capitalizing on them, and gratitude.  2. Session 2 focused on the skill of mindfulness.  3. In session 3, facilitators presented the skill of positive reappraisal.  4. Session 4 contained two skills: personal strengths and attainable goal setting.  5. In session 5, the focus was on acts of kindness.  6. In session 6, the final session of the intervention, facilitators worked with participants to plan continued practice of the skills and they provided suggestions for making engagement in the skills an ongoing habit. |
| **Patel, et al., 2023** | The course structure comprised six sections:  1. Welcome. Introductions, purpose of DAC course, confidentiality and peer learning  2. Module 1: What is dementia? Background of dementia and dementia in the UK, pathology, prevalence, and models of dementia  3. Module 2: Positive engagement. Psychological concepts and activities for positive communication and engagement (personhood, malignant social psychology, mental stimulation, promoting strengths, maximising potential)  4. Module 3: Caring for someone with dementia. Practical information on caring (activities of daily living, nutrition, risk management), services (non-drug treatments, CST, medication) and the effects of caring (impact, caregiver needs, signposting in the UK)  5. Reflections on course. Questions, feedback on course  6. Ending. Thank you for attending, closing of course |
| **Salehinejad et al., 2022** | **12 computer-interactive sessions covering the following sections:**  **1. Documents (text and images):** This section included four parts: (1) Information about dementia: this part contains five related topics, including understanding the disease, types of dementia, symptoms, diagnosis and the progression of the disease. (2) Behavioral disorders: the most frequent behavioral and psychological symptoms of dementia and their characteristics are described, and ways to cope with them are illustrated by example and practical advice. Anxiety, depression, aggressive behavior, sleep problems and hallucinations are some of the disorders discussed in this part. (3) Self-care for the caregivers: the session includes information and practical advice for maintaining and stimulating the relative’s balance and actions, such as managing stress, relaxing methods, make eating and sleeping well, remaining socially connected, social and financial support, asking and accepting help  and planning for the future. (4) Guides for better care: this part discussed routine care and looking after patients, such as feeding, bathing, dressing, home safety, patient transfer, enjoying meetings and holidays, driving, exercises and entertainment.  **2. Videos:** (a) relaxing training, (b) practical care training and (c) experiences of other caregivers.  **3. Important events and news:** Some information about new findings on dementia and what is happening worldwide. This section hosts news and blogs, websites, stories and books, conferences, and associations and support groups from across the field of dementia  **4. Forum:** an anonymous private platform for colleagues to communicate, share emotions, express concerns and find solutions to common problems. If necessary, a clinical psychologist participates in the discussion. |
| **Torkamani et al., 2014** | **Four key features:**  1. ‘ALADDIN TV’ provides information and educational material about dementia, as well as musical entertainment and relaxation and exercise techniques.  2. The ‘SOCIAL NETWORKING’ feature provides a forum for carers using ALADDIN to communicate with each other.  3. ‘MY TASKS’ is the distant monitoring feature of ALADDIN, where carers complete questionnaires about their own and their relatives’ health.  4. The ‘CONTACT US’ feature allows the carer to alert the clinical site and/or generate a request for contact. |
| **Windle et al., 2025** | **iSupport consists of five modules and 23 accompanying exercises:**  **MODULE 1 Introduction to dementia**  What is dementia and how does it affect someone  **MODULE 2 Being a carer**  The journey together  Improving communication  Supported decision-making  Involving others  **MODULE 3 Caring for me**  Reducing stress in everyday life  Making time for pleasant activities  Thinking differently  **MODULE 4 Providing everyday care**  Eating and drinking: more pleasant mealtimes  Eating, drinking and preventing health problems  Toileting and continence care  Personal care  An enjoyable day  **MODULE 5 Dealing with behaviour changes**  Introduction  Memory loss  Aggression  Depression, anxiety and apathy  Difficulty sleeping  Delusions and hallucinations  Repetitive behaviours  Walking and getting lost  Changes in judgement  Putting it all together |
| **Xiao et al., 2024** | **The intervention with carers was a facilitator-enabled online multicomponent programme, including psychoeducation using the online iSupport programme, online carer support groups and need-based access to information and services**  **1. Online iSupport program**  - Positive thoughts, meaningful interactions with and enabling self-determination for PWD (M2).  - Self-care & coping with stress (M3).  - Promoting functional ability, health and preventing complications for PWD (M4).  - Preventing and managing changed behaviours of PWD (M5).  - Access care services for carers and PWD (M1-5, M6).  **2. Carer peer support**  - Share knowledge, skills and experiences.  - Share information and resources about care services.  - Share and confirm positive thoughts.  - Gain emotional support.  - Gain social support.  **3. Access to information/services**  - Empower carers to use respite care.  - Link carers to dementia & aged care services for PWD.  - Link carers to health care services in need. |

Note: Terminology used in this table is based on the terminology presented by the respective authors in the referenced papers.

**Supplementary file 10: GRADE assessments**

| Certainty assessment | | | | | | | № of patients | | Effect | | Certainty | Importance |
| --- | --- | --- | --- | --- | --- | --- | --- | --- | --- | --- | --- | --- |
| № of studies | Study design | Risk of bias | Inconsistency | Indirectness | Imprecision | Other considerations | Online psychosocial intervention | placebo | Relative (95% CI) | Absolute (95% CI) |  |  |
| Burden score | | | | | | | | | | | | |
| 13 | randomized trials | serious | serious^a^ | not serious | serious^b^ | none | 340 | 265 | - | SMD 0.06 lower (0.25 lower to 0.12 higher) | ⨁⨁◯◯ Low |  |
| Self-efficacy score | | | | | | | | | | | | |
| 3 | randomized trials | very serious | not serious | not serious | not serious^c^ | none | 251 | 247 | - | SMD 0.20 higher (0.03 higher to 0.37 higher) | ⨁⨁◯◯ Low |  |

Note: CI: confidence interval; SMD: standardized mean difference

Explanations:

a. There was moderate heterogeneity across studies (I² = 47%, Chi² *P* = 0.03), with some variation in effect directions and confidence intervals that did not fully overlap.

b. Although the total sample size met the optimal information size, the confidence interval was wide and included both a small benefit and no effect. As the effect estimate was imprecise and crossed the threshold for decision-making, we downgraded the certainty of evidence due to imprecision.

c. The confidence interval did not include no effect and was entirely on the side of benefit. The effect estimate reached the minimally important difference (SMD = 0.20), suggesting a small but potentially meaningful effect. Although the total sample size did not fully meet the optimal information size, the precision was deemed sufficient; thus, we did not downgrade for imprecision.

**Supplementary file 11: PRISMA Checklist**

| **Section and Topic** | **Item #** | **Checklist item** | **Location where item is reported** |
| --- | --- | --- | --- |
| **TITLE** | | |  |
| Title | 1 | Identify the report as a systematic review. | Title page |
| **ABSTRACT** | | |  |
| Abstract | 2 | See the PRISMA 2020 for Abstracts checklist. | Abstract |
| **INTRODUCTION** | | |  |
| Rationale | 3 | Describe the rationale for the review in the context of existing knowledge. | Introduction |
| Objectives | 4 | Provide an explicit statement of the objective(s) or question(s) the review addresses. | Introduction |
| **METHODS** | | |  |
| Eligibility criteria | 5 | Specify the inclusion and exclusion criteria for the review and how studies were grouped for the syntheses. | “Inclusion and exclusion criteria” in the Methods section; Table 1 |
| Information sources | 6 | Specify all databases, registers, websites, organisations, reference lists and other sources searched or consulted to identify studies. Specify the date when each source was last searched or consulted. | “Search strategy” in the Methods section |
| Search strategy | 7 | Present the full search strategies for all databases, registers and websites, including any filters and limits used. | Supplemental File 6 |
| Selection process | 8 | Specify the methods used to decide whether a study met the inclusion criteria of the review, including how many reviewers screened each record and each report retrieved, whether they worked independently, and if applicable, details of automation tools used in the process. | “Study records and selection” in the Methods section |
| Data collection process | 9 | Specify the methods used to collect data from reports, including how many reviewers collected data from each report, whether they worked independently, any processes for obtaining or confirming data from study investigators, and if applicable, details of automation tools used in the process. | “Data extraction” in the Methods section |
| Data items | 10a | List and define all outcomes for which data were sought. Specify whether all results that were compatible with each outcome domain in each study were sought (e.g. for all measures, time points, analyses), and if not, the methods used to decide which results to collect. | “Data extraction” in the Methods section |
|  | 10b | List and define all other variables for which data were sought (e.g. participant and intervention characteristics, funding sources). Describe any assumptions made about any missing or unclear information. | “Data extraction” in the Methods section |
| Study risk of bias assessment | 11 | Specify the methods used to assess risk of bias in the included studies, including details of the tool(s) used, how many reviewers assessed each study and whether they worked independently, and if applicable, details of automation tools used in the process. | “Quality appraisal” in the Methods section |
| Effect measures | 12 | Specify for each outcome the effect measure(s) (e.g. risk ratio, mean difference) used in the synthesis or presentation of results. | “Data synthesis” in the Methods section |
| Synthesis methods | 13a | Describe the processes used to decide which studies were eligible for each synthesis (e.g. tabulating the study intervention characteristics and comparing against the planned groups for each synthesis (item #5)). | N/A |
|  | 13b | Describe any methods required to prepare the data for presentation or synthesis, such as handling of missing summary statistics, or data conversions. | N/A |
|  | 13c | Describe any methods used to tabulate or visually display results of individual studies and syntheses. | N/A |
|  | 13d | Describe any methods used to synthesize results and provide a rationale for the choice(s). If meta-analysis was performed, describe the model(s), method(s) to identify the presence and extent of statistical heterogeneity, and software package(s) used. | “Data synthesis” in the Methods section |
|  | 13e | Describe any methods used to explore possible causes of heterogeneity among study results (e.g. subgroup analysis, meta-regression). | N/A |
|  | 13f | Describe any sensitivity analyses conducted to assess robustness of the synthesized results. | “Data synthesis” in the Methods section |
| Reporting bias assessment | 14 | Describe any methods used to assess risk of bias due to missing results in a synthesis (arising from reporting biases). | N/A |
| Certainty assessment | 15 | Describe any methods used to assess certainty (or confidence) in the body of evidence for an outcome. | “Quality appraisal” in the Methods section |
| **RESULTS** | | |  |
|  | 16a | Describe the results of the search and selection process, from the number of records identified in the search to the number of studies included in the review, ideally using a flow diagram. | Supplemental File 1 |
|  | 16b | Cite studies that might appear to meet the inclusion criteria, but which were excluded, and explain why they were excluded. | N/A |
| Study characteristics | 17 | Cite each included study and present its characteristics. | Tables 2-5 |
| Risk of bias in studies | 18 | Present assessments of risk of bias for each included study. | “Quality appraisal” in the Result section; Figure 2-3 |
| Results of individual studies | 19 | For all outcomes, present, for each study: (a) summary statistics for each group (where appropriate) and (b) an effect estimate and its precision (e.g. confidence/credible interval), ideally using structured tables or plots. | Supplementary File 4-5 |
| Results of syntheses | 20a | For each synthesis, briefly summarise the characteristics and risk of bias among contributing studies. | “Quality appraisal” in the Result section |
|  | 20b | Present results of all statistical syntheses conducted. If meta-analysis was done, present for each the summary estimate and its precision (e.g. confidence/credible interval) and measures of statistical heterogeneity. If comparing groups, describe the direction of the effect. | “Effectiveness of online psychosocial interventions for caregivers of people with dementia” in Result section |
|  | 20c | Present results of all investigations of possible causes of heterogeneity among study results. | “Effectiveness of online psychosocial interventions for caregivers of people with dementia” in Result section |
|  | 20d | Present results of all sensitivity analyses conducted to assess the robustness of the synthesized results. | “Effectiveness of online psychosocial interventions for caregivers of people with dementia” in Result section |
| Reporting biases | 21 | Present assessments of risk of bias due to missing results (arising from reporting biases) for each synthesis assessed. | N/A |
| Certainty of evidence | 22 | Present assessments of certainty (or confidence) in the body of evidence for each outcome assessed. | “Quality appraisal” in the Result section |
| **DISCUSSION** | | |  |
| Discussion | 23a | Provide a general interpretation of the results in the context of other evidence. | Discussion |
|  | 23b | Discuss any limitations of the evidence included in the review. | Discussion |
|  | 23c | Discuss any limitations of the review processes used. | “Strengths and limitations” in Discussion section |
|  | 23d | Discuss implications of the results for practice, policy, and future research. | “Directions and areas for future work” in Discussion section |
| **OTHER INFORMATION** | | |  |
| Registration and protocol | 24a | Provide registration information for the review, including register name and registration number, or state that the review was not registered. | Methods |
|  | 24b | Indicate where the review protocol can be accessed, or state that a protocol was not prepared. | Methods |
|  | 24c | Describe and explain any amendments to information provided at registration or in the protocol. | N/A |
| Support | 25 | Describe sources of financial or non-financial support for the review, and the role of the funders or sponsors in the review. | Acknowledgments |
| Competing interests | 26 | Declare any competing interests of review authors. | Declaration of interests |
| Availability of data, code and other materials | 27 | Report which of the following are publicly available and where they can be found: template data collection forms; data extracted from included studies; data used for all analyses; analytic code; any other materials used in the review. | Supplemental files 7-10 |

*From:* Page MJ, McKenzie JE, Bossuyt PM, Boutron I, Hoffmann TC, Mulrow CD, et al. The PRISMA 2020 statement: an updated guideline for reporting systematic reviews. BMJ 2021;372:n71. doi: 10.1136/bmj.n71

For more information, visit: <http://www.prisma-statement.org/>
